# Effectiveness of sprint interval training in enhancing adolescent physical fitness: a systematic review and meta-analysis

**DOI:** 10.7717/peerj.21252

**Published:** 2026-06-02

**Authors:** Tiancheng Xu, Songpeng Su

**Affiliations:** 1 Guangzhou Sport University, Guangdong Province, China; 2 School of Athletic Training, Guangzhou Sport University, Guangdong Province, China

**Keywords:** Sprint interval training, SIT, Adolescent, Physical fitness, Physical training

## Abstract

**Background:**

Adolescents possess distinct physiological characteristics, including immature skeletal and neuromuscular development, which necessitate training modalities different from those designed for adults. Conventional training methods often present limitations in both efficiency and safety for this population.

**Objectives:**

This study aimed to quantify the effects of sprint interval training (SIT) on the physical fitness of adolescents, thereby providing evidence to support the optimization of training regimens for this demographic.

**Methods:**

PubMed, Web of Science, MEDLINE, and the Cochrane Library were searched systematically for eligible studies up to 2 June 2025. Study selection was performed according to the PICOS principle. Following data extraction, the Cochrane RoB 2.0 tool and the GRADE approach were employed to assess the risk of bias and the certainty of evidence, respectively. Data analysis was performed using Stata 18 software. Standardized mean differences (SMD) or mean differences (MD) with 95% confidence intervals were calculated as pooled effect estimates for all outcomes.

**Results:**

A total of 18 randomized controlled trials, involving a total of 565 participants, were included in the final analysis. The overall effects demonstrated that SIT significantly improves adolescents’ aerobic capacity (VO_2_max, MD = 3.66, *p* = 0.000), anaerobic capacity (peak power, MD = 48.39, *p* = 0.001; average power, MD = 36.64, *p* = 0.001), jumping ability (squat jump, MD = 1.23, *p* = 0.000; countermovement jump, MD = 1.74, *p* = 0.000; standing long jump, MD = 6.81, *p* = 0.025), body composition (body mass index, SMD = −0.63, *p* = 0.002; body fat percentage, SMD = −0.68, *p* = 0.000), sprint ability (10 m, MD = −0.16, *p* = 0.007; 20 m, MD = −0.15, *p* = 0.001; 30 m, MD = −0.22, *p* = 0.022) and change-of-direction ability (T test, MD = −0.27, *p* = 0.000).

**Conclusion:**

SIT exerts significant positive effects on adolescents’ aerobic capacity, anaerobic capacity, jumping ability, body composition, sprint ability, and change-of-direction ability. These findings support the use of SIT as an effective and time-efficient training method to enhance physical fitness in adolescents, with practical implications for both school physical education and athletic development programs. When implementing SIT, it is essential to adhere to the principles of adolescent physical development and apply individualized training protocols.

## Introduction

Adolescence is both a critical period for physical development and a key stage for establishing lifelong healthy behaviors (Guohai, Liu & Xiaojian, 2016; Viru et al., 1999). According to the World Health Organization (WHO), children and adolescents should accumulate at least 60 min per day, on average, of moderate-to-vigorous-intensity physical activity that is primarily aerobic (Bull et al., 2020). Unlike adults, adolescents possess distinct physiological characteristics. Their skeletal growth plates remain open, resulting in higher mechanical vulnerability (Caine, DiFiori & Maffulli, 2006). Concurrently, rapid limb growth often leads to a temporary lag in neuromuscular control, manifesting as decreased coordination and force production efficiency (Quatman-Yates et al., 2012). These fundamental differences dictate that adolescent training cannot simply be a scaled-down version of adult programs. Instead, it must prioritize movement quality and foundational neuromuscular control, with precisely managed training loads (Lloyd et al., 2016). Furthermore, physiological responses and the development of motor capacities differ between sexes during puberty. For instance, male adolescents typically demonstrate more pronounced gains in anaerobic capacity and muscular strength, whereas female adolescents often show greater advantages in flexibility and aerobic endurance development; these differences may further influence their responses and adaptations to training stimuli (Lloyd et al., 2015). Aerobic and anaerobic capacities, jumping ability, speed, and change-of-direction comprise the core dimensions for evaluating athletic potential and health status in youth (Lloyd et al., 2015). Emerging evidence indicates that performance constitutes a key determinant of self-esteem and psychological well-being during this developmental stage (Eime et al., 2013). In the general adolescent population, robust aerobic capacity underpins cardiorespiratory fitness and favorable metabolic health (Zheng, Xu & Zhang, 2025), whereas anaerobic capacity governs performance in rapid, high-intensity actions and is a principal driver of explosive power (Lloyd et al., 2015). Jumping, sprinting, and change-of-direction skills are foundational to most daily activities and sport-specific tasks (Lloyd et al., 2015). For young athletes, the importance of these attributes is magnified. Superior aerobic capacity provides sustained energy delivery in endurance-oriented sports (*e.g*., distance running, swimming) and safeguards technical–tactical execution under fatigue (Engel et al., 2018). Anaerobic capacity enables maximal force and velocity production in brief, explosive events such as sprinting and weightlifting. Jumping, sprinting, and change-of-direction directly determine movement efficiency in basketball jump-shooting (Erculj, Blas & BracIc, 2010), sprint starts (Vanderka et al., 2016), and soccer dribbling (Beato et al., 2018).

Traditional training methods aimed at enhancing these health and performance indicators in adolescents primarily include endurance training and resistance training. Endurance training, typically involving sustained aerobic exercise, primarily improves cardiorespiratory function and aerobic capacity (Armstrong & Barker, 2011). Resistance training, primarily utilizing external loads, aims to increase strength and power, thereby promoting the development of qualities like jumping ability, sprint ability, and change-of-direction (Faigenbaum et al., 2009). However, for the adolescent population, these conventional methods present several practical limitations, including long session durations, monotonous formats, high demands on training resources, and potential safety concerns. Consequently, there is a need for more efficient training modalities for adolescents that can concurrently develop various components of physical fitness.

Interval training is a classic method in adolescent physical training; by alternating high-intensity exercise bouts with low-intensity recovery periods, it can markedly enhance health status and cardiorespiratory endurance (Hall et al., 2023). A frequently investigated variant, sprint interval training (SIT), requires participants to perform “all-out” efforts at or above the power output (or velocity) associated with maximal oxygen uptake ( $\dot{\rm V}$O₂max) (Poon et al., 2024). Typically, each SIT bout lasts ≤ 30 s and is delivered *via* running or cycling (Poon et al., 2024; Ramos et al., 2015). These characteristics afford SIT the advantages of brief exercise duration, high mechanical power output, simple implementation, and pronounced physiological effects. Meta-analytic evidence supports the efficacy of SIT in improving both aerobic and anaerobic performance in healthy adults (Gist et al., 2014; Sloth et al., 2013) and athletes (Yang et al., 2021). Importantly, adolescents report greater enjoyment during high-intensity compared with moderate-intensity exercise (Song & Lan, 2024), suggesting that SIT may be particularly well-suited to this population. Nevertheless, adolescents’ maturational status and neuromuscular function differ markedly from those of adults, and training responses to SIT may therefore be influenced by additional factors. To date, findings in youth remain fragmented and inconclusive.

Given the unique characteristics of adolescent physical development and the pivotal role of SIT in enhancing physical fitness, this study aims to conduct a systematic review and meta-analysis. Using aerobic capacity, anaerobic capacity, body composition, jumping ability, sprint ability, and change-of-direction ability as outcome measures, we will comprehensively examine the effects of SIT on youth physical fitness, thereby providing an evidence-based reference for promoting adolescent health.

### Information and research methods

This systematic review and meta-analysis was conducted in strict accordance with the Preferred Reporting Items for Systematic Reviews and Meta-Analyses (PRISMA) guidelines and was prospectively registered with the International Prospective Register of Systematic Reviews (PROSPERO) (ID: CRD420251065085) (Page et al., 2021). The original protocol for this study was registered on 1 June 2025 and was revised on 11 August 2025 and 13 August 2025 in accordance with the actual search and screening processes. Both the original and revised versions are available on the PROSPERO website (https://www.crd.york.ac.uk/PROSPERO/recorddashboard).

### Search strategy

**Figure 1:**
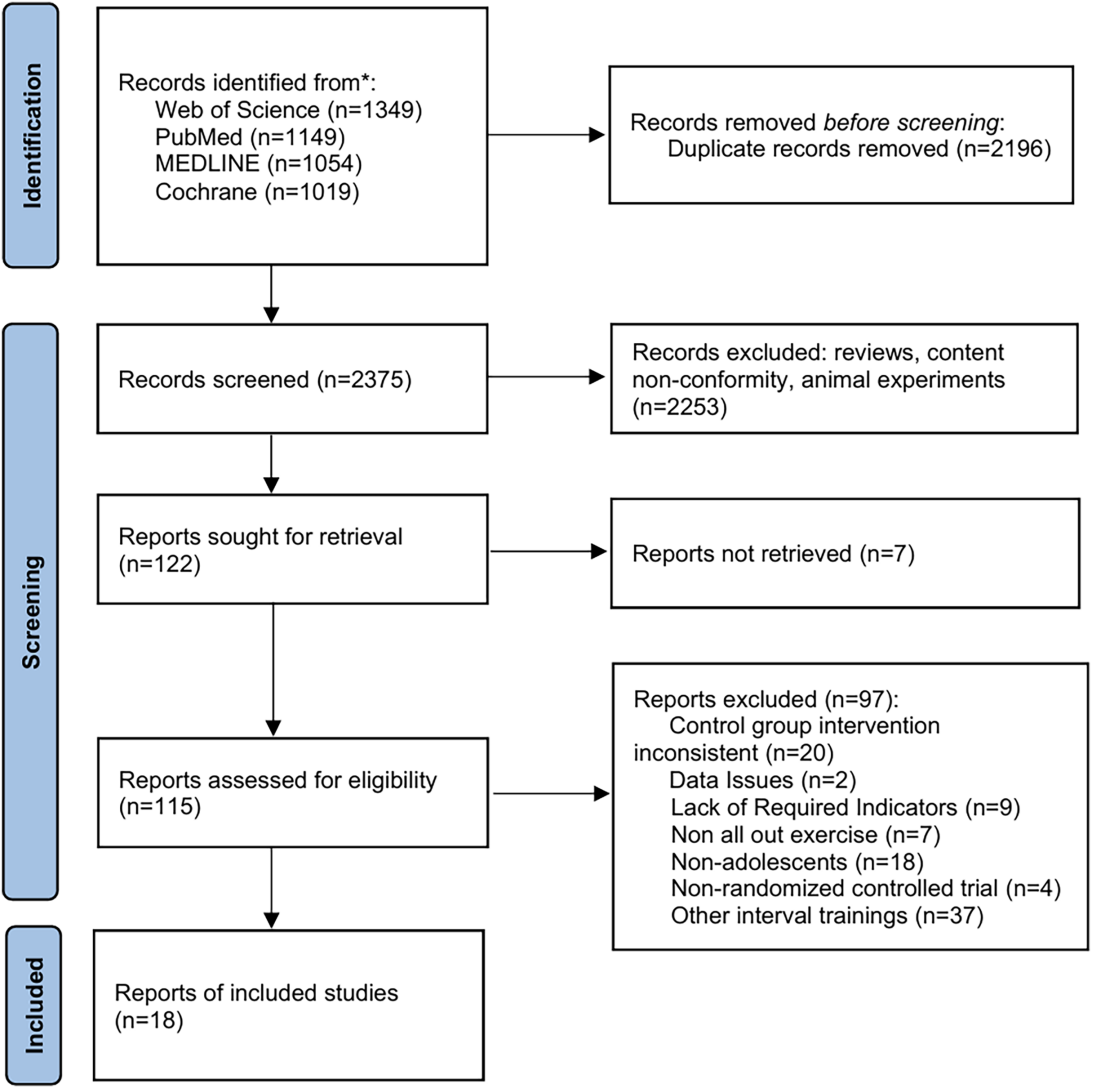
PRISMA flow chart for inclusion and exclusion of studies.

**Figure 2:**
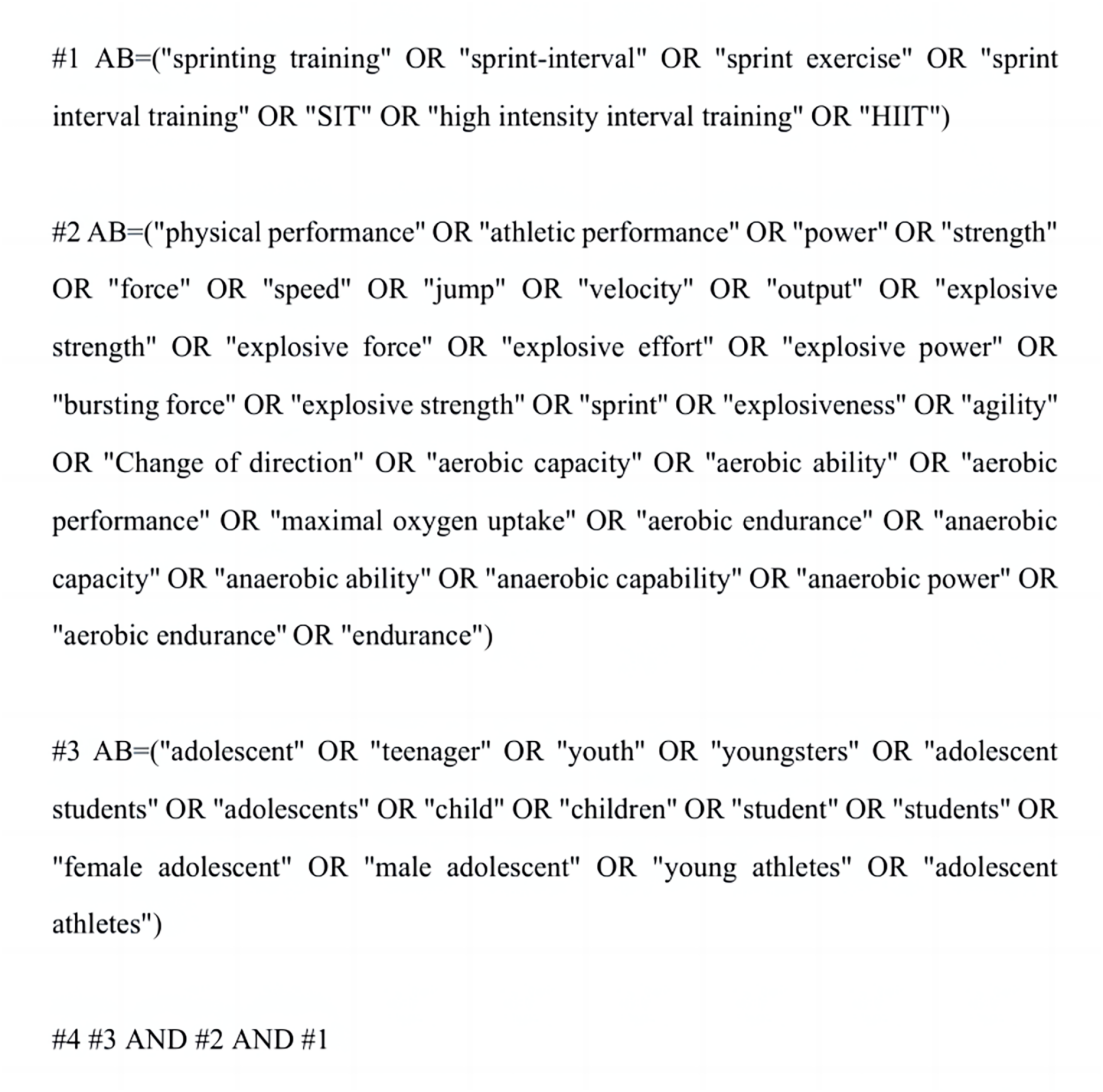
Web of Science literature selection strategy.

The search process was conducted in strict accordance with the PRISMA guidelines (Fig. 1). A pilot search was completed before registration (registration date: 1 June 2025), and the formal search was undertaken on 2 June 2025 across PubMed, Web of Science, MEDLINE, and the Cochrane Library, limited to English-language records. The search terms included “sprint interval training”, “SIT”, “physical performance”, “power”, “strength”, “speed”, “change-of-direction”, “aerobic capacity”, “anaerobic capacity”, “adolescent”, and “teenager”. The complete search process is illustrated in Fig. 2 (exemplified for Web of Science). Two investigators (TX, SS) independently designed and ran all searches; discrepancies were adjudicated by a third investigator (ZX). Citation tracking of included studies and related reviews was undertaken to capture additional records.

### Study selection

This study determined the inclusion criteria based on the Population, Intervention, Comparison, Outcomes, and Study design (PICOS) principle (Liberati et al., 2009).

The specific inclusion criteria were as follows: (1) P: Participants were adolescents aged 10–19 years (Plummer et al., 2017), free from known cardiovascular, musculoskeletal, or metabolic diseases that would preclude participation in high-intensity exercise. (2) I: The experimental group underwent SIT. The SIT protocol was defined as training performed on a cycle ergometer or *via* running; each sprint effort had a duration of ≤30 s; each sprint was required to be performed at an “all-out” or maximal intensity; and active or passive low-intensity recovery was employed between efforts (Poon et al., 2024). To ensure the intervention induced valid physiological adaptations and maintained continuity and appropriateness of the training stimulus, only studies with a total intervention duration of ≥4 weeks and a frequency of ≥2 sessions per week were included in this study (Haff & Triplett, 2021). (3) C: The control group received one of the following: regular sport-specific training, standard physical education classes, no training, or a wait-list control. All other conditions were maintained identical to the experimental group, with the exception of the SIT intervention. (4) Outcomes: direct indices of physical fitness, encompassing body composition markers—body mass index (BMI) and body fat percentage (BFP), jumping ability indices—squat jump (SJ), countermovement jump (CMJ), and standing long jump (SLJ), sprint ability indices—10, 20, and 30 m, change-of-direction ability—T-test, aerobic capacity—VO₂max, and anaerobic capacity—peak power (PP) and average power (AP). (5) S: Only randomized parallel-group controlled trials were included.

The exclusion criteria were as follows: (1) P: Participants outside the specified age range or diagnosed with conditions known to impair high-intensity exercise capacity. (2) I: Interventions that did not meet the defined SIT intensity or duration thresholds, or constituted other forms of interval training. (3) C: Control groups that implemented alternative training methods, or studies where baseline measurements showed significant differences between the experimental and control groups. (4) O: Unavailable full-text articles or studies failing to report means and standard deviations for the outcome measures. (5) S: Non randomized controlled trials (RCTs) or randomized crossover trials.

### Data extraction

The literature search was completed on 6 June 2025. After the search, all records were imported into Zotero 7 to remove duplicates. Two reviewers (TX and SS) then independently screened titles and abstracts to identify studies requiring full-text retrieval. The same reviewers subsequently extracted data into a standardized, piloted Microsoft Excel form. Extracted variables comprised: study characteristics (first author and year of publication), participant characteristics (sex, athletic status, mean age, and sample size), intervention characteristics (experimental and control protocols, duration, session length, and weekly frequency), and outcome data (outcome measures together with their means and standard deviations).

All pre- and post-intervention data were recorded as mean ± standard deviation and subsequently converted to change-from-baseline values ± standard deviation for meta-analytical purposes. Both the screening and data-extraction processes were performed independently by the two researchers (TX and SS); disagreements were resolved by a third senior researcher (ZX). When full texts or essential data were unavailable, the corresponding authors were contacted by e-mail (up to two reminders) to obtain the missing information.

### Bias risk and quality of evidence assessment

The risk of bias in the included studies was assessed using the Cochrane Risk of Bias tool (ROB 2.0). The assessment was conducted independently by two researchers (TX and SS), with any discrepancies resolved through consultation with a third researchers (ZX). The evaluation criteria encompassed five domains: bias arising from the randomization process, bias due to deviations from the intended interventions, bias due to missing outcome data, bias in the measurement of the outcome, and bias in the selection of the reported result. The overall risk of bias for each study was judged according to the following criteria: a study was rated ‘Low risk’ if all domains were judged as low risk; ‘High risk’ if at least one domain was judged as high risk; and ‘Some concerns’ if at least one domain was judged as some concerns.

The certainty of the evidence was graded with GRADE (Grading of Recommendations Assessment, Development and Evaluation) using GRADEpro GDT (Guyatt et al., 2011). For every outcome, two researchers (TX and SS) independently assessed risk of bias, inconsistency, indirectness, imprecision, and publication bias, rating the certainty as high, moderate, low, or very low. Discrepancies were adjudicated by a third researcher (ZX).

### Statistical analysis

Data analysis was performed using Stata 18.0 software. The primary effect parameters were calculated as the mean change from baseline ± standard deviation. For all outcomes, standardized mean differences (SMD) with 95% confidence intervals were used as pooled effect estimates when outcome units were inconsistent, while mean differences (MD) were applied when outcome units were consistent. The magnitude of SMD was interpreted using Cohen’s criteria, with values of 0.2, 0.5, and 0.8 representing small, medium, and large effect sizes, respectively (Cohen, 1988). For MD, statistical significance was determined by whether the 95% confidence intervals included zero.

The I² statistic was used to quantify heterogeneity among included studies, with I^2^ < 25% indicating negligible heterogeneity, 25% ≤ I^2^ ≤ 75% indicating moderate heterogeneity, and I² > 75% indicating high heterogeneity (Higgins et al., 2003). To account for potential heterogeneity, all effects were pooled using random-effects models (Higgins & Green, 2008). Where substantial heterogeneity was present (I^2^ ≥ 25%), sensitivity analyses using the leave-one-out method and meta-regression were performed to investigate potential sources. The meta-regression included seven covariates: sex (male, female, mixed), training status (athletes, non-athletes), obesity status (obese, non-obese), intervention frequency (<3/week, ≥3/week), intervention duration (<12 weeks, ≥12 weeks), SIT modality (running, cycling), and control group type (no training/usual activity, physical education/regular training). To ensure reliability and interpretability, meta-regression was conducted only for outcomes with more than 10 included RCTs (Chandler et al., 2019). Sensitivity analysis was performed to evaluate the robustness of the meta-analysis results. Statistical significance was set at *p* < 0.05.

Subgroup analyses were performed to examine the specific effects of SIT across different populations, based on training status (athletes, non-athletes) and obesity status (obese, non-obese). To maintain statistical power, subgroup analyses were conducted only for outcomes with more than 10 included studies (Chandler et al., 2019).

Publication bias was first examined by visual inspection of funnel plots and then quantified with Egger’s regression test; *p* < 0.05 indicated evidence of significant bias (Egger et al., 1997). If asymmetry was detected, the “trim-and-fill” method was applied to adjust the funnel plot, and the pooled effect was reassessed after imputation of potentially missing studies (Duval & Tweedie, 2000).

## Results

### Study characteristics

A total of 18 articles comprising 22 RCTs were ultimately included. The specific inclusion and exclusion process is illustrated in Fig. 1. The characteristics of the study participants are presented in Table 1, which was constructed according to the PICO framework: P: The experimental groups comprised a total of 309 subjects, while the control groups included 256 subjects. Participants were aged between 13 and 19 years, primarily consisting of adolescents who were obese, overweight, or soccer players. I: The intervention for the experimental groups was SIT in the form of running or cycling. Sprint durations ranged from 5 s to 30 s, with recovery intervals between 15 s and 4 min. The intervention periods varied from 4 to 15 weeks, with a frequency of 2–3 sessions per week. Each session lasted between 10 and 20 min. C: The control groups mostly engaged in regular activities or underwent no specific training. Apart from the intervention, all other conditions were consistent with the experimental groups. O: Outcome measures included BMI, BFP, 10 m, 20 m, 30 m, CMJ, SJ, SLJ, T-test, PP, AP and VO₂ max.

**Table 1:** Characteristics of study participants.

Studies	Participants	Sample size		Age		Experimental group				Control group	Key outcome indicators
		E	C	E	C	SIT protocols (Intensity)	Frequency	Duration		CON protocols	
Abassi et al. (2023)	Overweight/ Obese adolescents (F)	13	12	16.40 ± 1.20		30 s sprint/30 s recovery (100–110% MAS)	3/week	16–20 min/	12 weeks	Regular physical activity	BMI, BFP, 10 m, 20 m, 30 m, T Test, SJ, CMJ, SLJ
Sun et al. (2024)	Sedentary adolescents (F/M)	6	6	18.50 ± 0.30		1–4 weeks: 8 s sprint/24 s recovery. 5–8 weeks: 10 s sprint/30 s recovery. (All out)	3/week	20 min	8 weeks	Daily activities	BMI, BFP
Racil et al. (2016)	Obese adolescents (F)	23	19	16.60 ± 0.90	16.90 ± 1.00	30 s sprint/30 s recovery (100% VO_2_peak)	3/week	/	12 weeks	No training	BFP, SJ, CMJ, VO_2_max
Racil et al. (2013)	Obese adolescents (F)	11	12	15.60 ± 0.70	15.90 ± 1.20	30 s sprint/30 s recovery (100–110% MAS)	3/week	/	12 weeks	No training	BMI, BFP, VO_2_max
Abassi et al. (2020)	Overweight/ Obese adolescents (F)	8	8	16.50 ± 1.07	16.90 ± 1.64	30 s sprint/30 s recovery (100–110%MAS)	3/week	10 min	12 weeks	No training	BMI, BFP
Martin-Smith et al. (2019)	Healthy adolescents (F/M)	22	30	17.00 ± 0.30	16.80 ± 0.50	30 s sprint/30 s recovery (All out)	3/week	25–26 min	4 weeks	Regular PE classes	VO_2_max
Martin et al. (2015)	Healthy adolescents (F/M)	26	23	16.90 ± 0.30	16.80 ± 0.60	30 s sprint/30 s recovery (All out)	3/week	/	7 weeks	Regular PE classes	BMI, VO_2_max
Cao, Tang & Zou (2022)	Obese adolescents (M)	15	15	11.40 ± 0.80	11.00 ± 0.70	15 s sprint/15 s recovery (90–100% MAS)	3/week	11 min	12 weeks	No training	BMI, BFP, VO_2_max
Cao et al. (2022)	Obese students (F/M)	20	20	11.20 ± 0.70	10.90 ± 0.40	15 s sprint/15 s recovery (90–100% MAS)	3/week	18 min	12 weeks	Regular PE classes	BMI, BFP, VO_2_max
Chuensiri, Suksom & Tanaka (2018)	Obese adolescents (M)	11	15	11.10 ± 0.20	10.60 ± 0.30	20 s cycling/10 s recovery (170% PPO)	3/week	/	12 weeks	Sedentary control	BMI, BFP, VO_2_max
Michailidis et al. (2023)	Soccer players (M)	15	14	16.20 ± 0.40	16.50 ± 0.30	15 s sprint/15 s recovery (120% MAS)	2/week	12–20 min	4 weeks	Regular soccer training	BFP, 10 m, 30 m, CMJ, SJ
Boer et al. (2014)	Intellectually disabled adolescents (F/M)	17	14	18.00 ± 3.20	17.40 ± 2.04	15 s cycling/45 s recovery (100–110%VT)	2/week	20 min	15 weeks	Daily activities	BFP, VO_2_max, BMI
Seo et al. (2019) (1:2)	Taekwondo players (M)	12	11	16.70 ± 0.84		30 s sprint/60 s recovery (90–100% HRmax)	2–3/week	/	4 weeks	Regular taekwondo training	VO_2_max, PP, AP, T Test
Seo et al. (2019) (1:4)	Taekwondo players (M)	12	11	16.70 ± 0.84		30 s sprint/120 s recovery (90–100% HRmax)	2–3/week	/	4 weeks	Regular taekwondo training	VO_2_max, PP, AP, T Test
Seo et al. (2019) (1:8)	Taekwondo players (M)	12	11	16.70 ± 0.84		30 s sprint/240 s recovery (90–100% HRmax)	2–3/week	/	4 weeks	Regular taekwondo training	VO_2_max, PP, AP, T Test
Ferley, Scholten & Vukovich (2020) (INC)	Soccer players (F/M)	17	15	15.71 ± 1.30	16.03 ± 1.17	6–30 s sprint/24–120 s recovery (100% MAS)	2/week	/	8 weeks	Regular physical activity	CMJ, SLJ
Ferley, Scholten & Vukovich (2020) (LEV)	Soccer players (F/M)	14	15	15.14 ± 1.08	16.03 ± 1.17	6 or 30 s sprint/24 or 120 s recovery (110–138% MAS)	2/week	/	8 weeks	Regular physical activity	CMJ, SLJ
Abassi et al. (2022)	Overweight/Obese adolescents (F)	13	12	16.40 ± 1.00		30 s sprint/30 s recovery (100–110% MAS)	3/week	/	12 weeks	No training	BMI, BFP
Derakhti et al. (2022) (RST)	Soccer players (M)	8	6	15.70 ± 0.50		20 m sprint/180 s recovery (All out)	2/week	/	4 weeks	Regular soccer training	10 m, 20 m, 30 m, CMJ, SLJ
Derakhti et al. (2022) (UST)	Soccer players (M)	10	6	15.70 ± 0.50		20 m sprint/180 s recovery (All out)	2/week	/	4 weeks	Regular soccer training	10 m, 20 m, 30 m, CMJ, SLJ
Salus et al. (2022)	Obese adolescents (M)	14	14	13.10 ± 0.30	13.70 ± 0.40	30 s cycling/240 s recovery (All out)	3/week	/	12 weeks	No training	BFP, VO_2_max
Xu et al. (2024)	Basketball players	10	10	16.10 ± 0.90	16.20 ± 0.60	5 s sprint/20 s recovery (All out)	3/week	/	6 weeks	Regular basketball training	VO_2_max, PP, AP, 20 m, T Test, SJ, CMJ

**Note:**

E, experimental group; C, control group; F, Female; M, Male; SIT, sprint interval training; CON, Control; MAS, maximal aerobic speed; PPO, peak power output; VT, ventilatory threshold; HRmax, Maximum Heart Rate; PE, physical education; BMI, Body Mass Index; BFP, Body Fat Percentage; PP, peak power; AP, average power; SJ, squat Jump; CMJ, countermovement jump; SLJ, standing long jump; a/b, the same author published two articles in the same year; 1:2, SIT work-to-rest ratio is 1:2; 1:4, SIT work-to-rest ratio is 1:4; 1:8, SIT work-to-rest ratio is 1:8; INC, SIT based on incline; LEV, SIT based on incline; RST, resisted sprint training; UST, unresisted sprint training.

### Bias risk and quality of evidence

**Figure 3:**
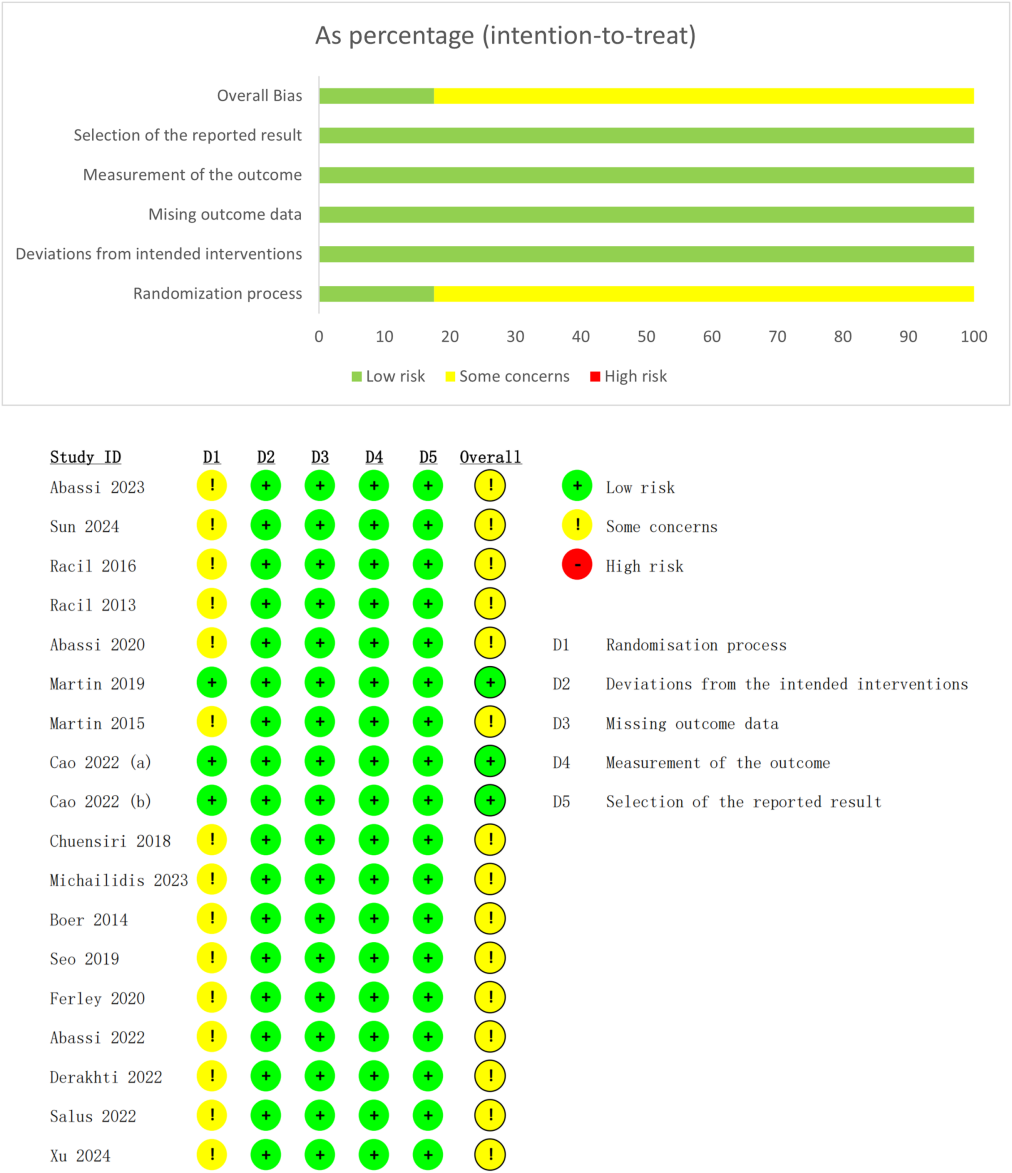
Risk of bias assessment chart.

This study employed the ROB 2.0 to assess the quality of the included literature. Overall, three studies (17.6%) were rated as ‘Low risk’, while the remaining 15 studies (82.4%) were rated as having ‘Some concern’. All included studies clearly described random sequence generation; however, only three studies mentioned allocation concealment procedures. Consequently, the randomization process domain was predominantly judged as having some concerns. The other four domains were overall rated as ‘Low risk’. This indicates that the study results possess relatively high internal validity and reliability (Fig. 3).

The assessment of all evidence quality using the GRADE online tool revealed that the quality of evidence for explosive power was moderate. The quality of evidence for body composition, change-of-direction, anaerobic capacity, and sprint ability was ‘Low’, and the quality for aerobic capacity was ‘Very Low’ (Table 2). These ratings were primarily influenced by issues related to allocation concealment, blinding, the small number of included studies, and substantial heterogeneity among the studies.

**Table 2:** GRADE summary of evidence.

Certainty assessment							No of patients		Effect		Certainty	Importance
No of studies	Study design	Risk of bias	Inconsistency	Indirectness	Imprecision	Other considerations	(EXP)	(CON)	Relative (95% CI)	Absolute (95% CI)		
Body composition												
24	RT	Serious^a^	Serious^b^	Not serious	Not serious	None	322	314	–	SMD 0.66 SD lower (0.91 lower to 0.41 lower)	⊕⊕〇〇 Low^a,b^	IMPORTANT
Change of direction ability												
5	RT	Serious^a^	Not serious	Not serious	Serious^c^	None	59	55	–	MD 0.27 s lower (0.41 lower to 0.13 lower)	⊕⊕〇〇 Low^a,c^	IMPORTANT
Aerobic capacity												
13	RT	Serious^a^	Very serious^d^	Not serious	Not serious	None	213	201	–	MD 3.66 ml/kg/min higher (2.67 higher to 4.65 higher)	⊕〇〇〇 Very low^a,d^	CRITICAL
Anaerobic capacity												
8	RT	Serious^a^	Not serious	Not serious	Serious^e^	None	62	86	–	MD 37.36 W higher (22.3 higher to 52.42 higher)	⊕⊕〇〇 Low^a,e^	CRITICAL
Sprint ability												
12	RT	Serious^a^	Serious^f^	Not serious	Not serious	None	133	110	–	MD 0.17 s lower (0.24 lower to 0.11 lower)	⊕⊕〇〇 Low^a,f^	IMPORTANT
Explosive power												
17	RT	Serious^a^	Not serious	Not serious	Not serious	None	233	206	–	MD 1.55 cm higher (1.16 higher to 1.95 higher)	⊕⊕⊕〇 Moderate^a^	CRITICAL

**Note:**

RT, randomised trials; CI, confidence interval; MD, mean difference; SMD, standardised mean difference; a, Most studies lacked allocation concealment or blinding; b, I^2^ = 55.9%; c, Only 5 RCTs were included; d, I^2^ = 82.4%; e, Only 8 RCTs were included; f, I^2^ = 48.5%; Risk of bias includes issues such as incorrect randomization, lack of allocation concealment, absence of blinding, excessive loss to follow-up, selective outcome reporting, or early termination of the study after observing a beneficial effect. If most of the studies exhibit one or more important risks of bias, the quality of evidence should be downgraded by one level; Inconsistency refers to considerable heterogeneity among studies or a very low *p*-value in heterogeneity tests. When substantial and unexplained inconsistency is present, the quality of evidence should be downgraded by one level. Indirectness arises when the patients, interventions, comparisons, or outcome measures in the available studies differ from the target PICO elements specified in the review question. If an important degree of indirectness exists, the quality of evidence should be downgraded by one level. Imprecision includes situations with a small sample size, a limited number of included studies, or confidence intervals that include the line of no effect. When evidence is uncertain due to an insufficient sample size or number of events, the quality of evidence should be downgraded by one level. Other considerations include publication bias, a large magnitude of effect, or a dose-response gradient. If there is a strong suspicion of publication bias, the quality of evidence should be downgraded by one level.

### Meta-analysis results

#### Aerobic capacity

**Figure 4:**
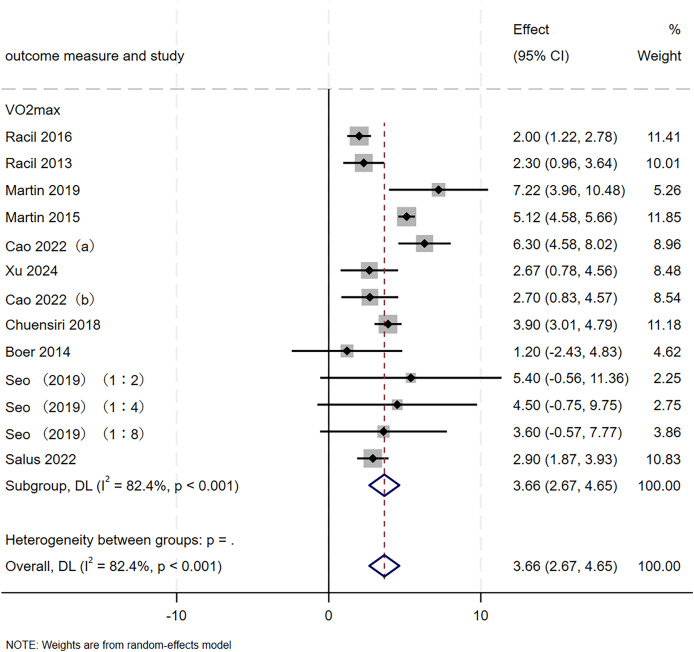
Forest plot of aerobic capacity. a/b, the same author published two articles in the same year.

A total of 13 RCTs, involving 388 participants, were included in this study to evaluate the effect of SIT on aerobic capacity in adolescents. As illustrated in Fig. 4, the meta-analysis revealed a significant improvement in aerobic capacity following SIT (MD = 3.66, 95% CI [2.67–4.65], *p* < 0.001). However, heterogeneity was substantial (I² = 82.4%, *p* < 0.001). Sequential removal of the trial by Martin et al. (2015) reduced I^2^ to 67.6% (*p* < 0.001) without materially altering the pooled estimate (MD = 3.41, 95% CI [2.51–4.30]), indicating that the original finding for aerobic capacity was robust (Table 3).

**Table 3:** Results of stepwise exclusion method.

Study	Heterogeneity within subgroups after removal	MD (SMD) within subgroups after removal	Overall heterogeneity	Overall MD (SMD)
Aerobic capacity (VO_2_max)				
Racil et al. (2016)	/	/	I^2^ = 73.9%, *p* < 0.001	3.86 (2.92, 4.80)
Racil et al. (2013)	/	/	I^2^ = 82.5%, *p* < 0.001	3.81 (2.76, 4.86)
Martin-Smith et al. (2019)	/	/	I^2^ = 82.8%, *p* < 0.001	3.46 (2.46, 4.46)
Martin et al. (2015)	/	/	I^2^ = 67.6%, *p* < 0.001	3.41 (2.51, 4.30)
Cao, Tang & Zou (2022)	/	/	I^2^ = 81.7%, *p* < 0.001	3.39 (2.38, 4.40)
Xu et al. (2024)	/	/	I^2^ = 83.5%, *p* < 0.001	3.75 (2.70, 4.80)
Cao et al. (2022)	/	/	I^2^ = 83.5%, *p* < 0.001	3.75 (2.70, 4.80)
Chuensiri, Suksom & Tanaka (2018)	/	/	I^2^ = 83.9%, *p* < 0.001	3.64 (2.50, 4.79)
Boer et al. (2014)	/	/	I^2^ = 83.4%, *p* < 0.001	3.77 (2.76, 4.78)
Seo et al. (2019) (1:2)	/	/	I^2^ = 83.8%, *p* < 0.001	3.62 (2.61, 4.62)
Seo et al. (2019) (1:4)	/	/	I^2^ = 83.9%, *p* < 0.001	3.63 (2.62, 4.64)
Seo et al. (2019) (1:8)	/	/	I^2^ = 83.9%, *p* < 0.001	3.66 (2.64, 4.68)
Salus et al. (2022)	/	/	I^2^ = 83.0%, *p* < 0.001	3.75 (2.65, 4.85)
Body composition (BMI)				
Abassi et al. (2023)	I^2^ = 64.9%, *p* = 0.004	−0.64 (−1.10, −0.19)	I^2^ = 61.4%, *p* < 0.001	−0.66 (−0.94, −0.38)
Sun et al. (2024)	I^2^ = 63.5%, *p* = 0.005	−0.68 (−1.11, −0.25)	I^2^ = 60.7%, *p* < 0.001	−0.68 (−0.95, −0.40)
Racil et al. (2013)	I^2^ = 64.8%, *p* = 0.004	−0.66 (−1.10, −0.21)	I^2^ = 61.3%, *p* < 0.001	−0.67 (−0.95, −0.39)
Abassi et al. (2020)	I^2^ = 64.9%, *p* = 0.004	−0.65 (−1.09, −0.21)	I^2^ = 61.4%, *p* < 0.001	−0.66 (−0.94, −0.38)
Martin et al. (2015)	I^2^ = 62.8%, *p* = 0.006	−0.69 (−1.15, −0.23)	I^2^ = 60.3%, *p* < 0.001	−0.68 (−0.96, −0.40)
Cao, Tang & Zou (2022)	I^2^ = 0.0%, *p* = 0.908	−0.45 (−0.70, −0.19)	I^2^ = 41.0%, *p* = 0.027	−0.57 (−0.80, −0.35)
Cao et al. (2022)	I^2^ = 63.4%, *p* = 0.005	−0.60 (−1.06, −0.15)	I^2^ = 61.0%, *p* < 0.001	−0.65 (−0.93, −0.36)
Chuensiri, Suksom & Tanaka (2018)	I^2^ = 64.2%, *p* = 0.004	−0.67 (−1.12, −0.22)	I^2^ = 60.0%, *p* < 0.001	−0.67 (−0.95, −0.39)
Boer et al. (2014)	I^2^ = 62.5%, *p* = 0.006	−0.69 (−1.14, −0.25)	I^2^ = 60.1%, *p* < 0.001	−0.68 (−0.96, −0.40)
Abassi et al. (2022)	I^2^ = 64.9%, *p* = 0.004	−0.64 (−1.10, −0.19)	I^2^ = 61.4%, *p* < 0.001	−0.66 (−0.94, −0.38)
Body composition (BFP)				
Abassi et al. (2023)	I^2^ = 65.3%, *p* = 0.001	−0.68 (−0.94, −0.38)	I^2^ = 61.5%, *p* < 0.001	−0.66 (−0.94, −0.38)
Sun et al. (2024)	I^2^ = 64.2%, *p* = 0.002	−0.71 (−1.11, −0.32)	I^2^ = 60.9%, *p* < 0.001	−0.68 (−0.95, −0.40)
Racil et al. (2016)	I^2^ = 46.5%, *p* = 0.044	−0.56 (−0.90, −0.23)	I^2^ = 51.9%, *p* = 0.003	−0.59 (−0.85, −0.34)
Racil et al. (2013)	I^2^ = 58.3%, *p* = 0.008	−0.60 (−0.97, −0.23)	I^2^ = 57.3%, *p* < 0.001	−0.61 (−0.88, −0.35)
Abassi et al. (2020)	I^2^ = 65.1%, *p* = 0.001	−0.67 (−1.07, −0.26)	I^2^ = 61.3%, *p* < 0.001	−0.65 (−0.93, −0.37)
Cao, Tang & Zou (2022)	I^2^ = 63.7%, *p* = 0.002	−0.64 (−1.04, −0.23)	I^2^ = 60.3%, *p* < 0.001	−0.64 (−0.91, −0.36)
Cao et al. (2022)	I^2^ = 65.2%, *p* = 0.001	−0.69 (−1.11, −0.27)	I^2^ = 61.4%, *p* < 0.001	−0.66 (−0.95, −0.38)
Chuensiri, Suksom & Tanaka (2018)	I^2^ = 44.5%, *p* = 0.055	−0.80 (−1.12, −0.47)	I^2^ = 52.6%, *p* = 0.003	−0.72 (−0.97, −0.46)
Michailidis et al. (2023)	I^2^ = 60.9%, *p* = 0.004	−0.74 (−1.13, −0.35)	I^2^ = 59.4%, *p* < 0.001	−0.69 (−0.97, −0.41)
Boer et al. (2014)	I^2^ = 65.1%, *p* = 0.001	−0.69 (−1.11, −0.28)	I^2^ = 61.4%, *p* < 0.001	−0.66 (−0.95, −0.38)
Abassi et al. (2022)	I^2^ = 65.3%, *p* = 0.001	−0.68 (−1.09, −0.27)	I^2^ = 61.5%, *p* < 0.001	−0.66 (−0.94, −0.38)
Salus et al. (2022)	I^2^ = 65.3%, *p* = 0.001	−0.67 (−1.09, −0.26)	I^2^ = 61.4%, *p* < 0.001	−0.66 (−0.94, −0.37)
Sprint ability (10 m)				
Abassi et al. (2023)	I^2^ = 64.3%, *p* = 0.061	−0.15 (−0.28, −0.01)	I^2^ = 52.9%, *p* = 0.020	−0.17 (−0.24, −0.10)
Michailidis et al. (2023)	I^2^ = 24.6%, *p* = 0.265	−0.10 (−0.26, −0.06)	I^2^ = 46.5%, *p* = 0.044	−0.16 (−0.24, −0.08)
Derakhti et al. (2022) (RST)	I^2^ = 63.8%, *p* = 0.063	−0.14 (−0.33, −0.05)	I^2^ = 53.0%, *p* = 0.019	−0.17 (−0.24, −0.10)
Derakhti et al. (2022) (UST)	I^2^ = 0.0%, *p* = 0.838	−0.22 (−0.26, −0.17)	I^2^ = 39.9%, *p* = 0.083	−0.17 (−0.25, −0.13)
Sprint ability (30 m)				
Abassi et al. (2023)	I^2^ = 31.5%, *p* = 0.232	−0.13 (−0.26, −0.00)	I^2^ = 28.7%, *p* = 0.172	−0.16 (−0.21, −0.10)
Michailidis et al. (2023)	I^2^ = 53.9%, *p* = 0.071	−0.29 (−0.47, −0.10)	I^2^ = 41.6%, *p* = 0.071	−0.19 (−0.25, −0.10)
Derakhti et al. (2022) (RST)	I^2^ = 82.3%, *p* = 0.003	−0.19 (−0.44, −0.05)	I^2^ = 51.5%, *p* = 0.024	−0.17 (−0.23, −0.10)
Derakhti et al. (2022) (UST)	I^2^ = 82.5%, *p* = 0.003	−0.25 (−0.47, −0.03)	I^2^ = 51.5%, *p* = 0.024	−0.18 (−0.24, −0.11)

**Note:**

a/b, the same author published two articles in the same year; 1:2, SIT work-to-rest ratio is 1:2; 1:4, SIT work-to-rest ratio is 1:4; 1:8, SIT work-to-rest ratio is 1:8; INC, SIT based on incline; LEV, SIT based on incline; RST, resisted sprint training; UST, unresisted sprint training.

#### Anaerobic capacity

**Figure 5:**
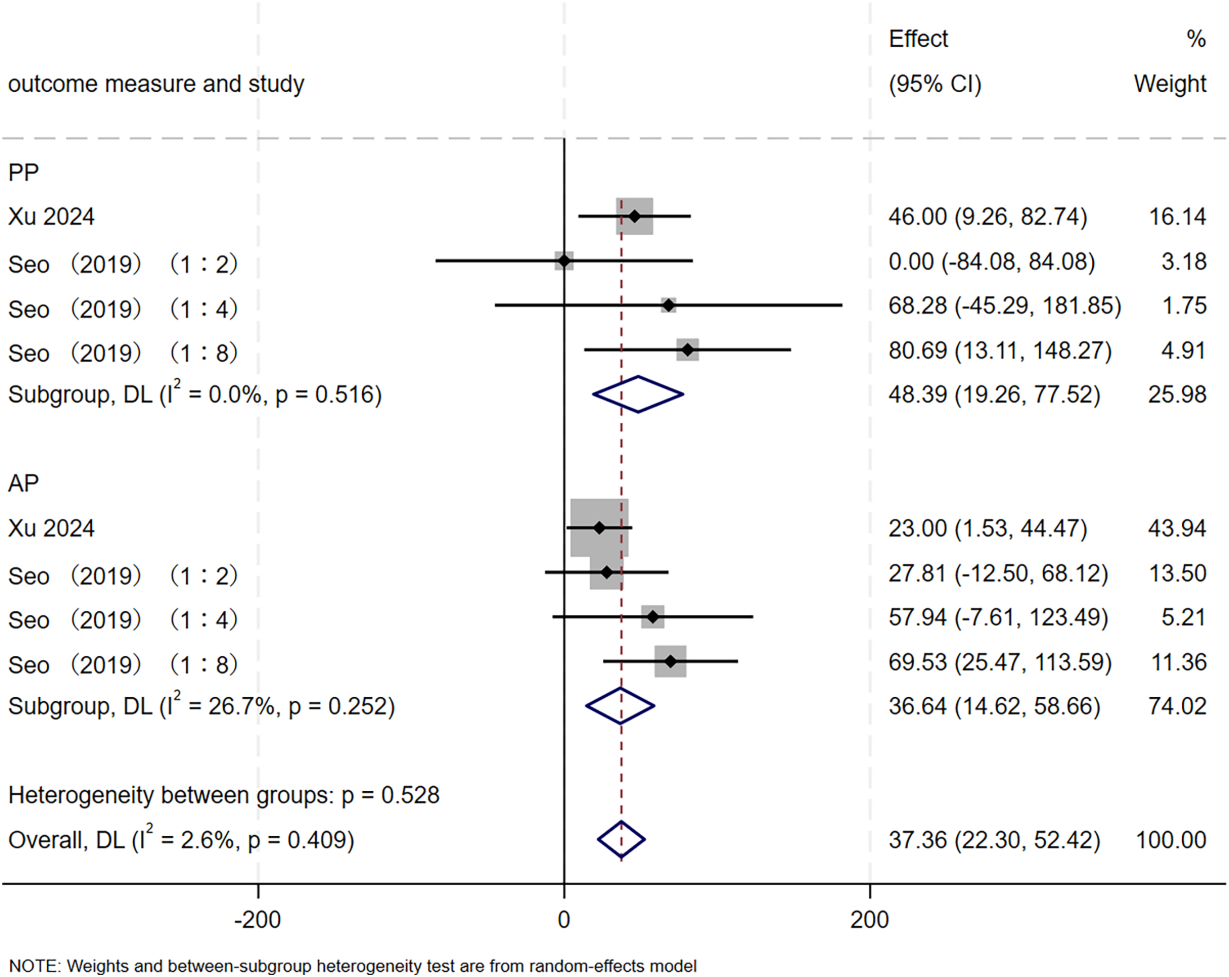
Forest plot of anaerobic capacity. 1:2, SIT work-to-rest ratio is 1:2; 1:4, SIT work-to-rest ratio is 1:4; 1:8, SIT work-to-rest ratio is 1:8.

This study included 8 RCTs involving 67 participants to evaluate the effect of SIT on anaerobic capacity in adolescents. As shown in Fig. 5, the pooled estimate indicated that SIT elicited a significant and positive improvement in anaerobic capacity (MD = 37.36, 95% CI [22.30–52.42], *p* < 0.001), with minimal heterogeneity observed across studies (I² = 2.6%, *p* = 0.409). Subgroup analyses revealed that SIT significantly increased both PP (MD = 48.39, 95% CI [19.26–77.52], *p* = 0.001) and AP (MD = 36.64, 95% CI [14.62–58.66], *p* = 0.001). No heterogeneity was detected for PP (I² = 0.0%, *p* = 0.516), whereas moderate heterogeneity was present for AP (I² = 26.7%, *p* = 0.252).

#### Jumping ability

**Figure 6:**
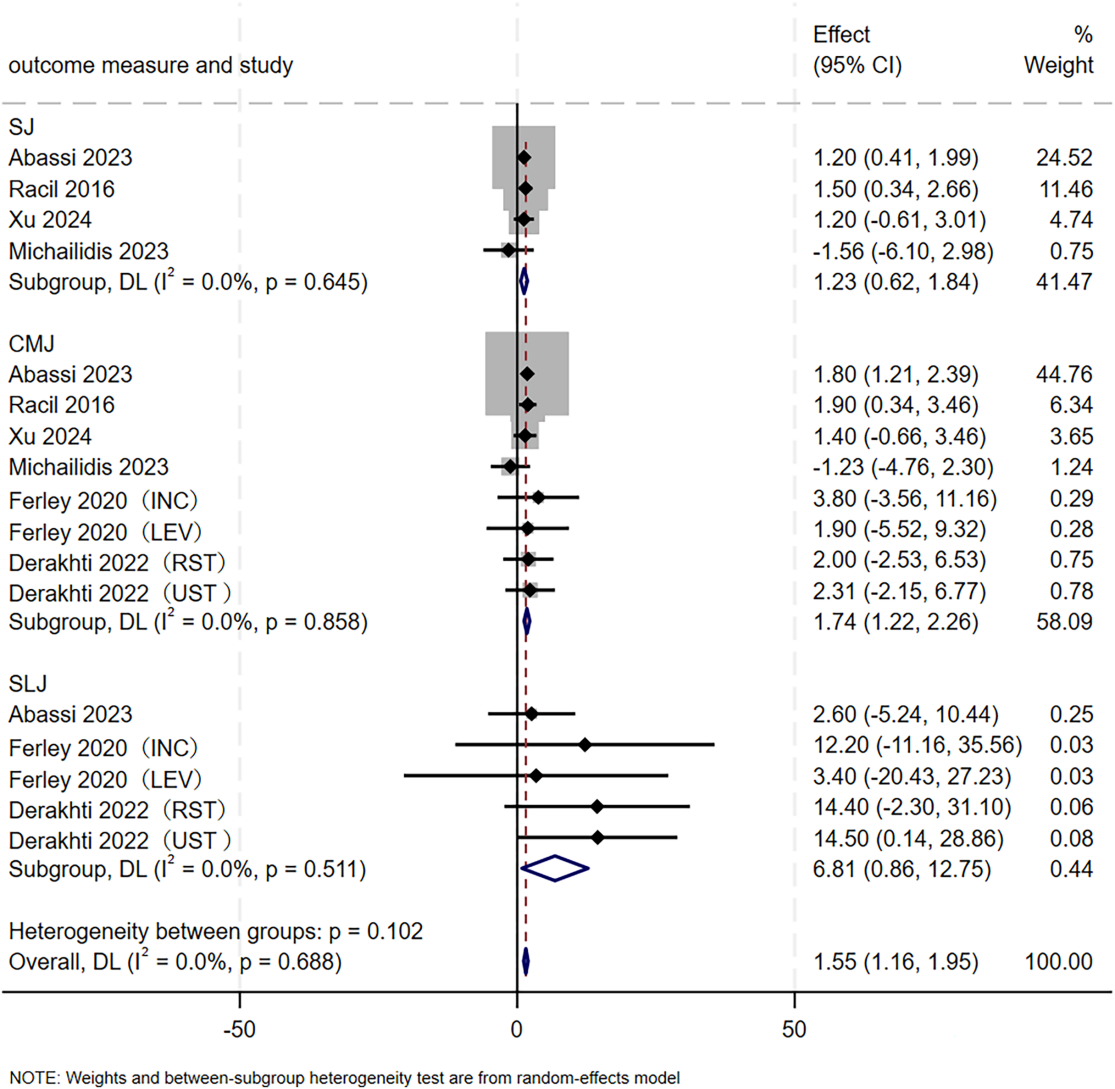
Forest plot of jumping ability. INC, SIT based on incline; LEV, SIT based on incline; RST, resisted sprint training; UST, unresisted sprint training.

A total of 17 RCTs, involving 187 participants, were included to evaluate the effect of SIT on jumping ability in adolescents. As shown in Fig. 6, the overall analysis revealed a significant positive improvement in jumping ability following SIT (MD = 1.55, 95% CI [1.16–1.95], *p* < 0.001), with no evidence of heterogeneity (I² = 0.0%, *p* = 0.688). Subgroup analyses further demonstrated that SIT significantly enhanced SJ (MD = 1.23, 95% CI [0.62–1.84], *p* < 0.001), CMJ (MD = 1.74, 95% CI [1.22–2.26], *p* < 0.001), and SLJ (MD = 6.81, 95% CI [0.86–12.75], *p* = 0.025) performance. No heterogeneity was observed for SJ (I² = 0.0%, *p* = 0.645), CMJ (I² = 0.0%, *p* = 0.858), and SLJ (I² = 0.0%, *p* = 0.511).

#### Body composition

**Figure 7:**
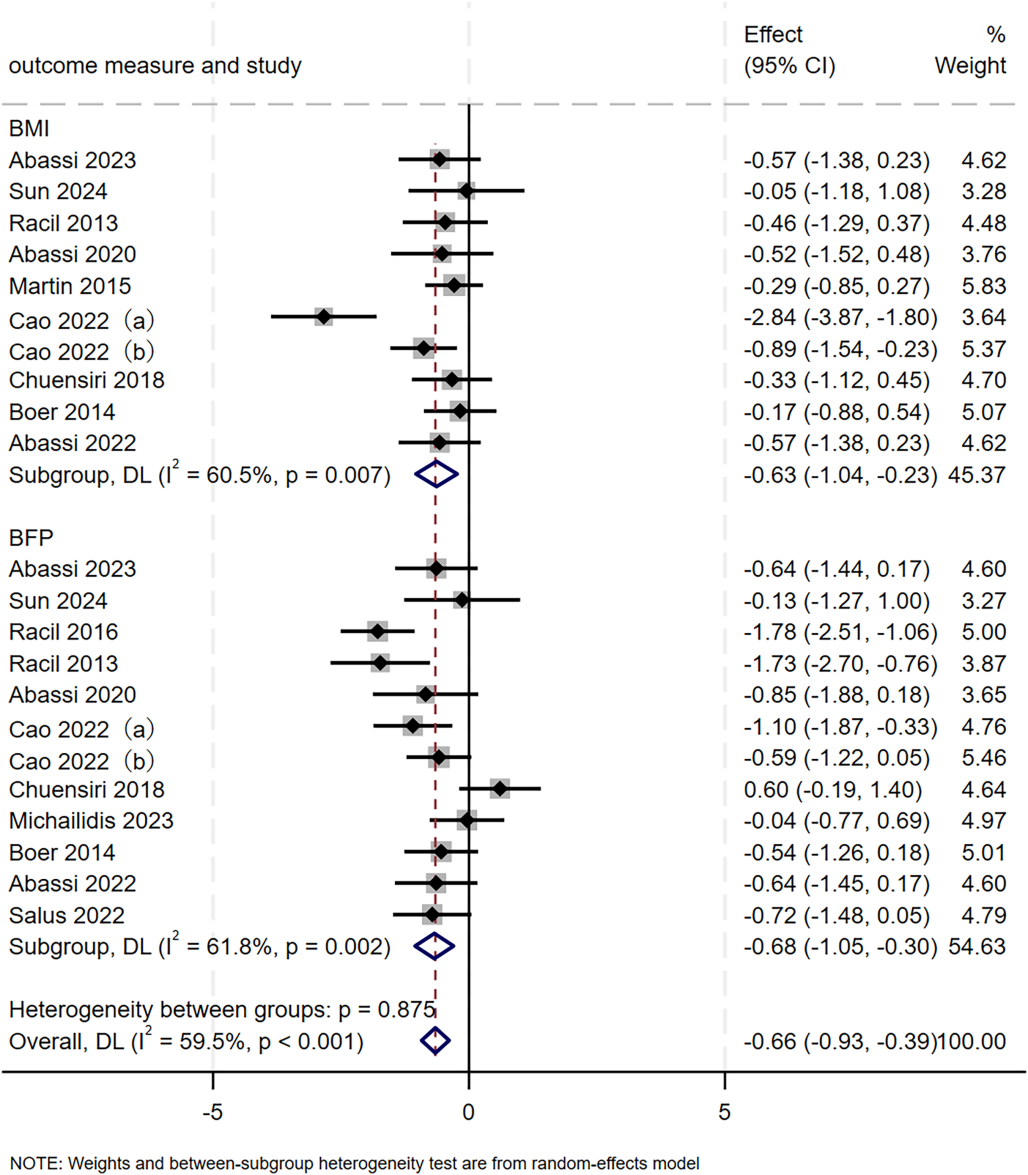
Forest plot of body composition. a/b, the same author published two articles in the same year.

A total of 22 RCTs, involving 377 participants, were included to evaluate the effect of SIT on body composition in adolescents. As illustrated in Fig. 7, the pooled estimate revealed a significant, favourable improvement (SMD = −0.66, 95% CI [−0.93 to −0.39], *p* < 0.001) with moderate heterogeneity (I² = 59.5%, *p* < 0.001). Subgroup analyses further revealed that SIT significantly reduced both BMI (SMD = −0.63, 95% CI [−1.04 to −0.23], *p* = 0.002) and BFP (SMD = −0.68, 95% CI [−1.05 to −0.30], *p* < 0.001). Moderate heterogeneity was observed within both the BMI (I² = 60.5%, *p* = 0.007) and BFP (I² = 61.8%, *p* = 0.002) subgroups. A leave-one-out analysis demonstrated that after excluding the study by Cao et al. (2022), heterogeneity within the BMI subgroup dropped to 0.0% (*p* = 0.908) and overall heterogeneity decreased to 41.0% (*p* < 0.001), while the pooled estimate remained significantly favorable (SMD = −0.63, 95% CI [−1.04 to −0.23], *p* = 0.002). Similarly, removal of the study by Chuensiri, Suksom & Tanaka (2018) reduced heterogeneity within the BFP subgroup to 44.5% (*p* = 0.055) and overall heterogeneity to 52.6%, yet the pooled effect remained significantly improved (SMD = −0.72, 95% CI [−0.97 to −0.46]). These findings indicated that the original results for body composition were robust (Table 3).

#### Sprint ability

**Figure 8:**
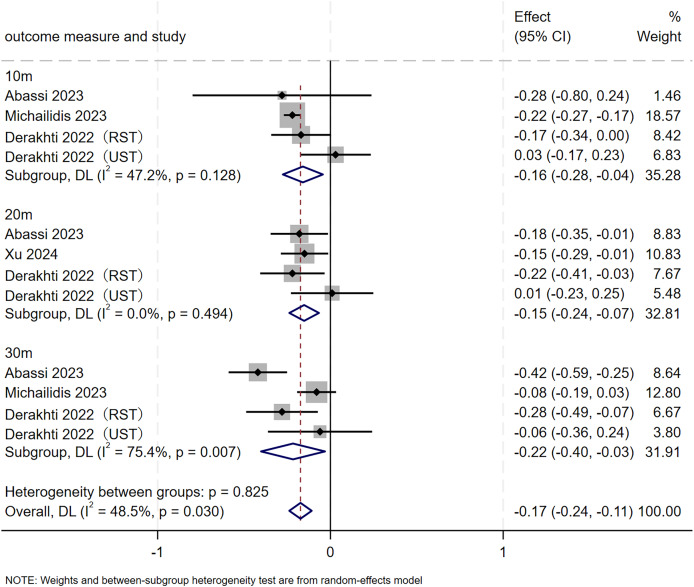
Forest plot of sprint ability. RST, resisted sprint training; UST, unresisted sprint training.

This study included 12 RCTs involving 98 participants to evaluate the effect of SIT on sprint ability in adolescents. As depicted in Fig. 8, the meta-analysis revealed a significant, favorable improvement in overall sprint ability (MD = −0.17, 95% CI [−0.24 to −0.11], *p* < 0.001), with moderate heterogeneity (I² = 48.5%, *p* = 0.030). Subgroup analyses showed that SIT significantly reduced 10 m (MD = −0.16, 95% CI [−0.28 to −0.04], *p* = 0.007), 20 m (MD = −0.15, 95% CI [−0.24 to −0.07], *p* = 0.001), and 30 m (MD = −0.22, 95% CI [−0.40 to −0.03], *p* = 0.022) sprint times. Heterogeneity was moderate for 10 m (I² = 47.2%, *p* = 0.128) and 30 m (I² = 75.4%, *p* = 0.007), but negligible for 20 m (I² = 0.0%, *p* = 0.494). A leave-one-out analysis revealed that after excluding the study by Derakhti et al. (2022), heterogeneity within the 10 m subgroup decreased to 0.0% (*p* = 0.838) and overall heterogeneity declined to 39.9% (*p* = 0.083), yet the pooled estimate remained significant (MD = −0.17, 95% CI [−0.25 to −0.13]). Likewise, removal of the study by Abassi et al. (2023) reduced heterogeneity within the 30 m subgroup to 31.5% (*p* = 0.232) and overall heterogeneity to 28.7% (*p* = 0.172), while the pooled effect remained significant (MD = −0.16, 95% CI [−0.21 to −0.10]). These findings indicated that the original results for sprint ability were robust (Table 3).

#### Change-of-direction ability

**Figure 9:**
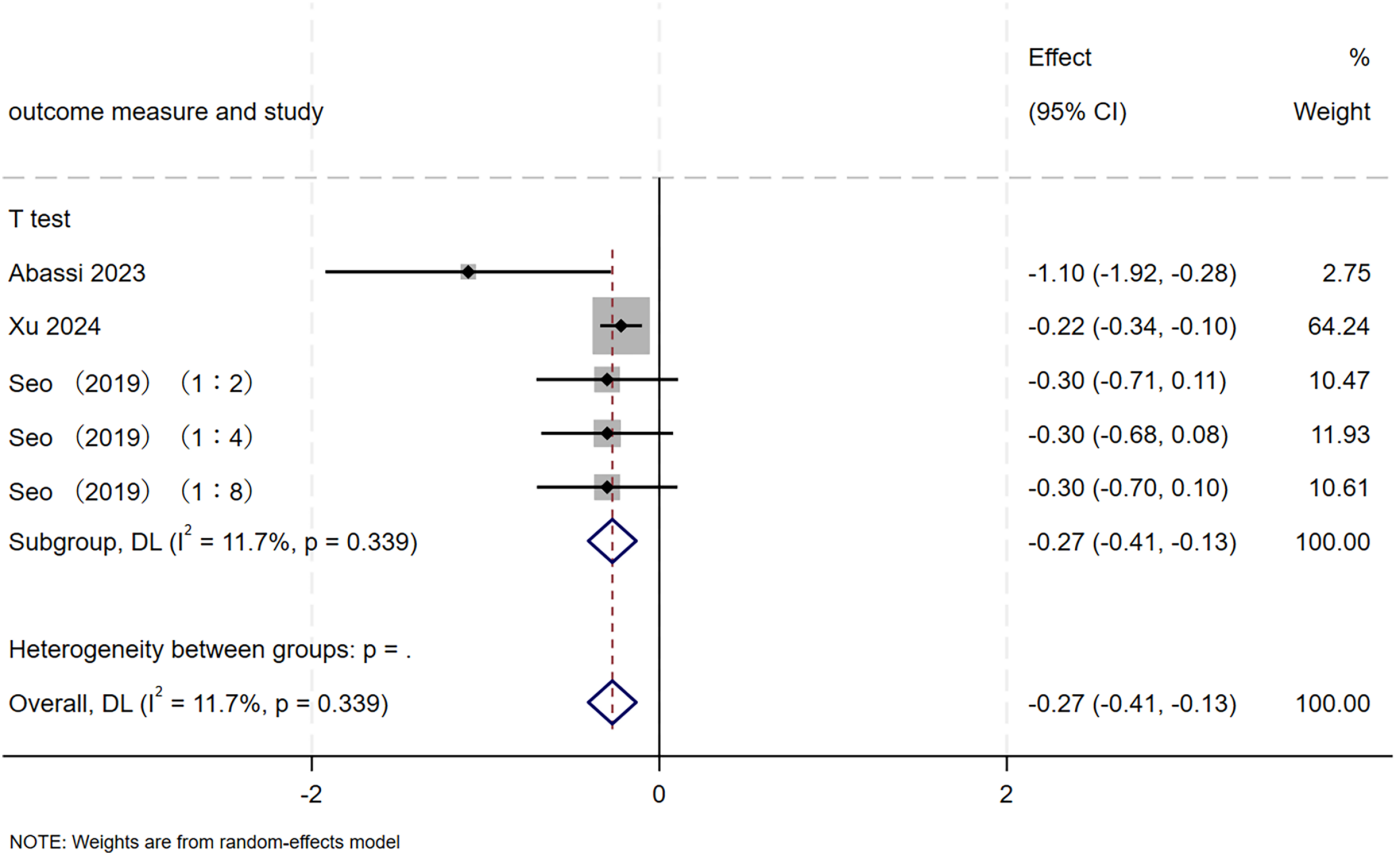
Forest plot of change-of-direction ability. RST, resisted sprint training; UST, unresisted sprint training.

A total of 5 RCTs, involving 92 participants, were included in this study to evaluate the effect of SIT on change-of-direction ability in adolescents. As shown in Fig. 9, the meta-analysis revealed a significant improvement in change-of-direction following SIT (MD = −0.27, 95% CI [−0.41 to −0.13], *p* < 0.001), with low heterogeneity across studies (I² = 11.7%, *p* = 0.339).

### Results of meta-regression

As shown in Table 4, meta-regression analyses were performed for aerobic capacity, body composition, and sprint ability. For aerobic capacity, regression analyses were conducted for sex, training status, obesity status, intervention frequency, intervention period, SIT type, and control group intervention type. The results indicated that improvements in VO₂ max were significantly associated with sex (*p* = 0.008), obesity status (*p* = 0.041), SIT type (*p* = 0.035), and control group intervention type (*p* = 0.026). No significant associations were found for the other covariates. The model explained 96.97% of the between-study heterogeneity, with a residual I² = 0.00%. For body composition, regression analyses were conducted for sex, obesity status, intervention frequency, intervention period, SIT type, and control group intervention type. The results showed that improvements in BMI and BFP were significantly associated with SIT type (*p* = 0.026). No significant associations were observed for the other covariates. The model explained 31.44% of the between-study heterogeneity, with a residual I² = 50.00%. For sprint ability, regression analyses were conducted for training status and intervention frequency (obesity status was automatically excluded from the model due to collinearity with other variables). The results revealed that these two variables did not significantly influence the overall heterogeneity in sprint ability. The model explained 2.57% of the between-study heterogeneity, with a residual I² = 48.91%.

**Table 4:** Results of meta regression.

	Coefficient (95% CI)	t	*p*	I^2^-residual	Adjusted R^2^
Aerobic capacity (*n* = 13)					
Gender	−4.21 [–6.76 to –1.67]	−4.26	0.008	0.00%	96.97%
Exercise status	2.54 [−0.17 to 5.25]	2.41	0.061		
Obesity status	12.98 [0.81–25.15]	2.74	0.041		
Intervention frequency	6.81 [−0.02 to 13.64]	2.56	0.050		
Intervention duration	10.46 [−2.01 to 22.93]	2.16	0.083		
Type of SIT	−2.84 [−5.37 to –0.30]	−2.88	0.035		
Control group intervention type	−4.83 [–8.81 to –0.84]	−3.11	0.026		
Body composition (*n* = 22)					
Gender	0.14 [−0.23 to 0.51]	0.79	0.440	50.00%	31.44%
Obesity status	0.52 [−0.68 to 1.72]	0.92	0.372		
Intervention frequency	0.17 [−0.74 to 1.08]	0.40	0.692		
Intervention duration	−0.29 [−1.46 to 0.87]	−0.54	0.598		
Type of SIT	0.94 [0.13 to 1.75]	2.48	0.026		
Control group Intervention type	−0.19 [–1.00 to 0.62]	−0.50	0.622		
Sprint ability (*n* = 12)					
Exercise status	−0.15 [−0.44 to 0.15]	−1.12	0.291	48.91%	2.57%
intervention frequency	−0.01 [−0.26 to 0.25]	−0.07	0.944		

### Subgroup analysis

As shown in Table 5, subgroup analyses were performed for aerobic capacity, jumping ability, body composition, and sprint ability. Regarding training status, the results indicated that, based on effect sizes, the improvements in aerobic capacity, jumping ability, body composition, and sprint ability were all greater in non-athletes compared to athletes. However, the magnitude of these effects did not differ significantly between the subgroups (*p* > 0.05). Regarding obesity status, the results indicated that the improvements in jumping ability, body composition, and sprint ability were greater in obese individuals compared to non-obese individuals. A significant difference in effect size between these subgroups was observed for body composition (*p* = 0.018), but not for the other two outcomes (*p* > 0.05). In contrast, for aerobic capacity, the improvement was greater in non-obese individuals compared to obese individuals, although the difference in effect size between these subgroups was not statistically significant (*p* > 0.05).

**Table 5:** Results of subgroup analysis.

	*n*	Heterogeneity within subgroups	SMD within subgroups (95% CI)	Overall heterogeneity	Overall SMD (95% CI)	Subgroup differences
Aerobic ability (exercise status)						
Athlete	4	I^2^ = 0.0%, *p* = 0.779	3.16 [1.58–4.74], *p* = 0.000	I^2^ = 82.4%, *p* < 0.001	3.66 [3.67–4.65], *p* = 0.000	0.594
Non-athlete	9	I^2^ = 88.0%, *p* < 0.001	3.69 [2.55–4.82], *p* = 0.000			
Aerobic ability (obesity status)						
Obese	6	I^2^ = 80.4%, *p* < 0.001	3.27 [2.19–4.34], *p* = 0.000	I^2^ = 82.4%, *p* < 0.001	3.66 [2.67–4.65], *p* = 0.000	0.288
Non-obese	7	I^2^ = 51.3%, *p* = 0.055	4.25 [2.79–5.71], *p* = 0.000			
Jumping ability (exercise status)						
Athlete	12	I^2^ = 0.0%, *p* = 0.467	1.21 [0.11–2.32], *p* = 0.032	I^2^ = 0.0%, *p* = 0.688	1.55 [1.16–1.95], *p* = 0.000	0.519
Non-athlete	5	I^2^ = 0.0%, *p* = 0.800	1.60 [1.18–2.02], *p* = 0.000			
Jumping ability (obesity status)						
Obese	5	I^2^ = 0.0%, *p* = 0.800	1.60 [1.18–2.02], *p* = 0.000	I^2^ = 0.0%, *p* = 0.000	1.55 [1.16–1.95], *p* = 0.000	0.519
Non-obese	12	I^2^ = 0.0%, *p* = 0.467	1.21 (0.11–2.32), *p* = 0.032			
Body composition (obesity status)						
Obese	18	I^2^ = 62.2%, *p* < 0.001	–0.77 [−1.07 to −0.46], *p* = 0.000	I^2^ = 59.5%, *p* < 0.001	–0.66 [–0.93 to −0.39], *p* = 0.000	0.018
Non-obese	4	I^2^ = 0.0%, *p* = 0.951	–0.17 [−0.56 to 0.22], *p* = 0.388			
Sprint ability (exercise status)						
Athlete	9	I^2^ = 42.4%, *p* = 0.085	–0.15 [−0.21 to −0.08], *p* = 0.000	I^2^ = 48.5%, *p* = 0.030	–0.17 [−0.24 to −0.11], *p* = 0.000	0.133
Non-athlete	3	I^2^ = 49.2%, *p* = 0.139	–0.30 [−0.48 to −0.11], *p* = 0.002			
Sprint ability (obesity status)						
Obese	3	I^2^ = 49.2%, *p* = 0.139	–0.30 [−0.48 to −0.11], *p* = 0.002	I^2^ = 48.5%, *p* = 0.030	–0.17 [−0.024 to −0.11], *p* = 0.000	0.133
Non-obese	9	I^2^ = 42.4%, *p* = 0.085	–0.15 [−0.21 to −0.08], *p* = 0.000			

### Publication bias

**Figure 10:**
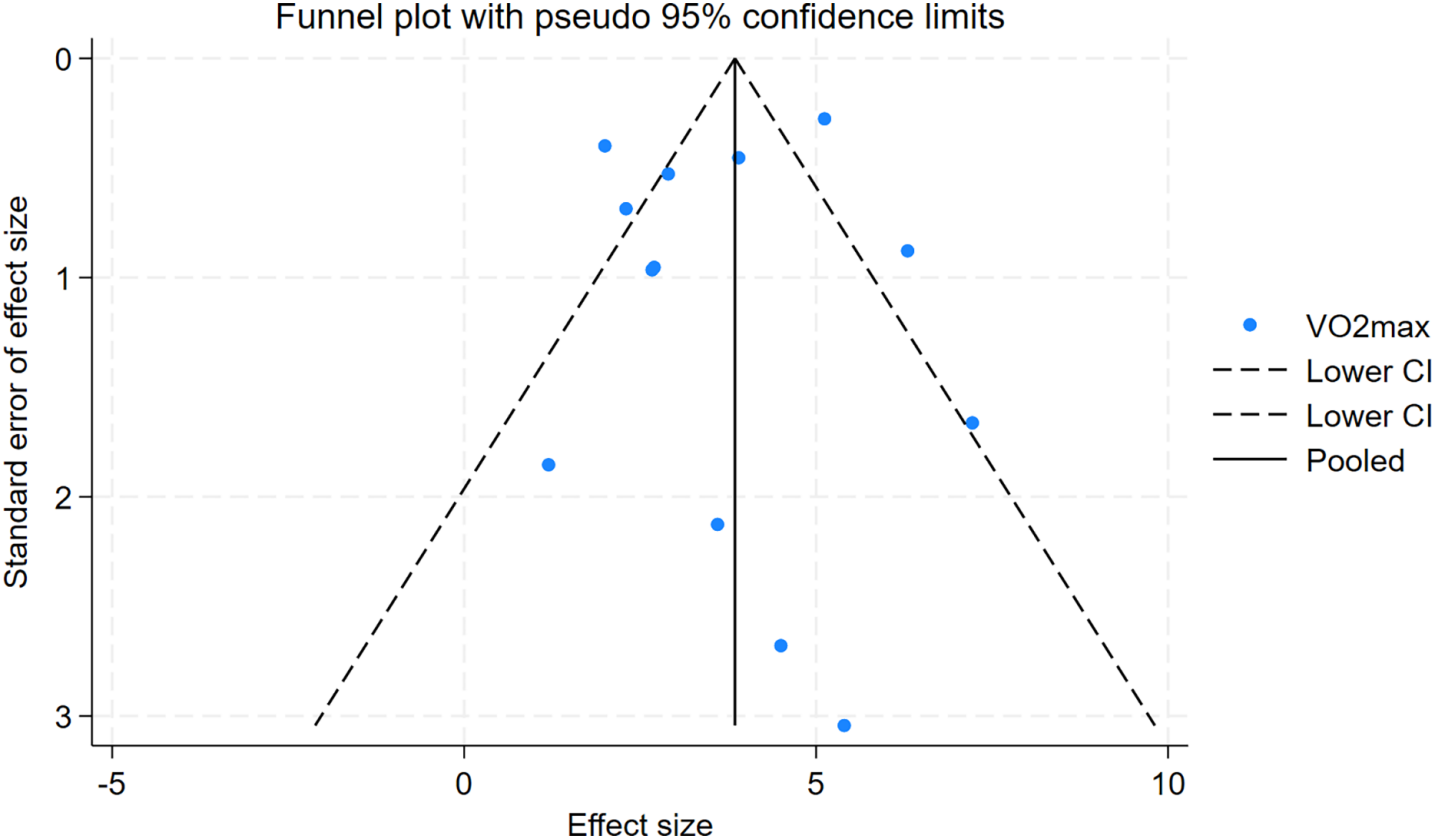
Funnel plots of aerobic capacity.

**Figure 11:**
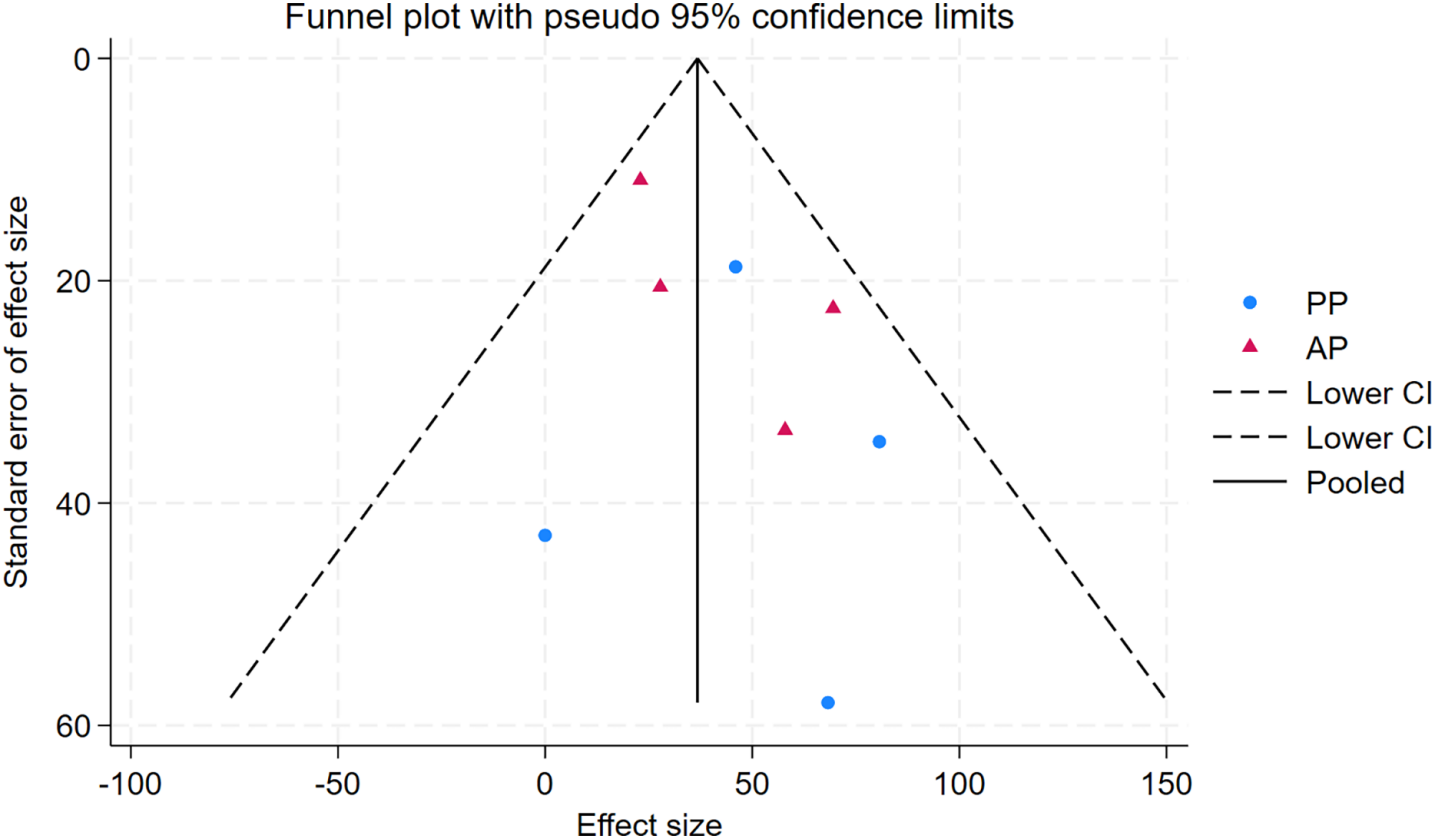
Funnel plot of anaerobic capacity.

**Figure 12:**
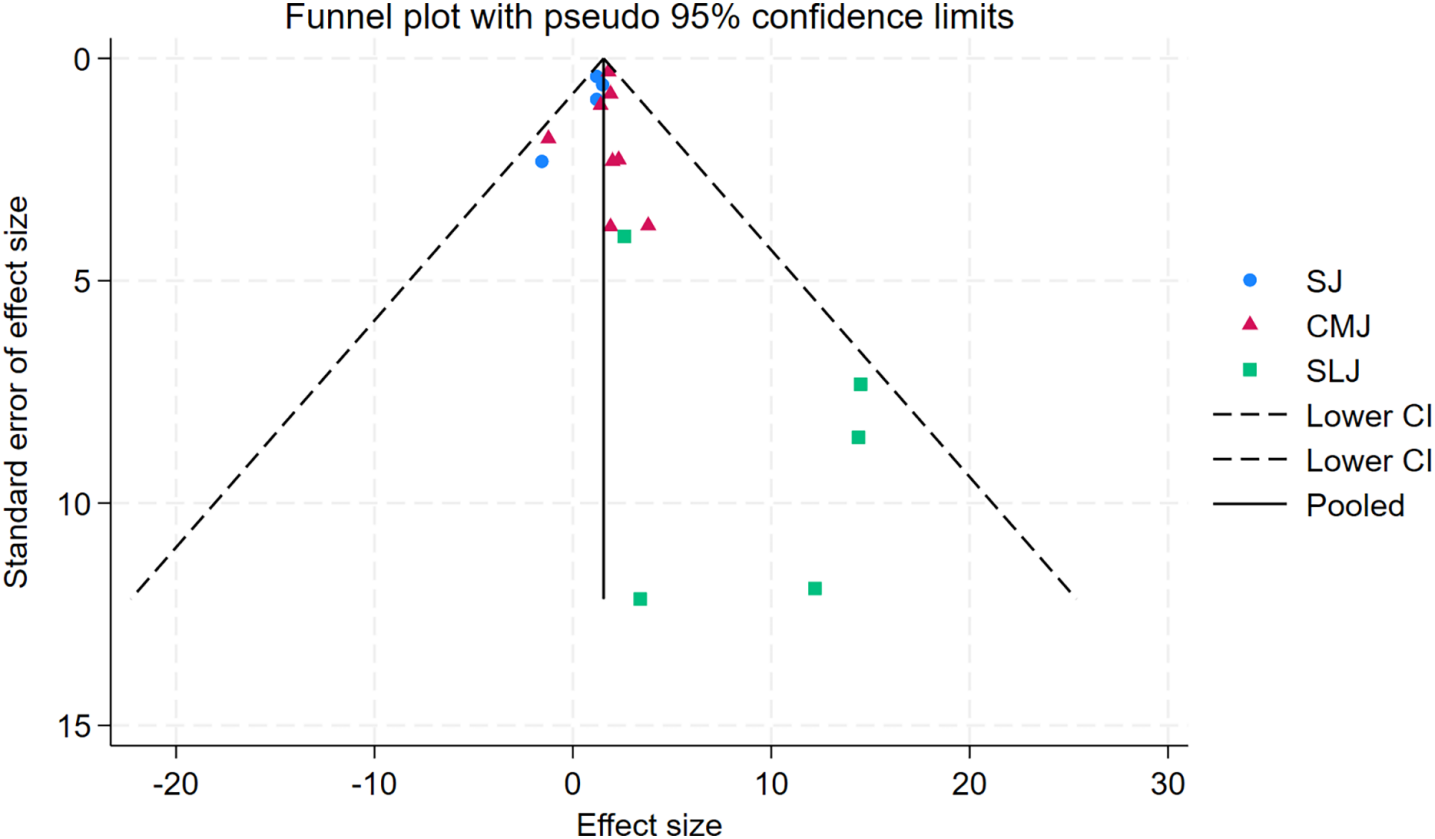
Funnel plot of jumping ability.

**Figure 13:**
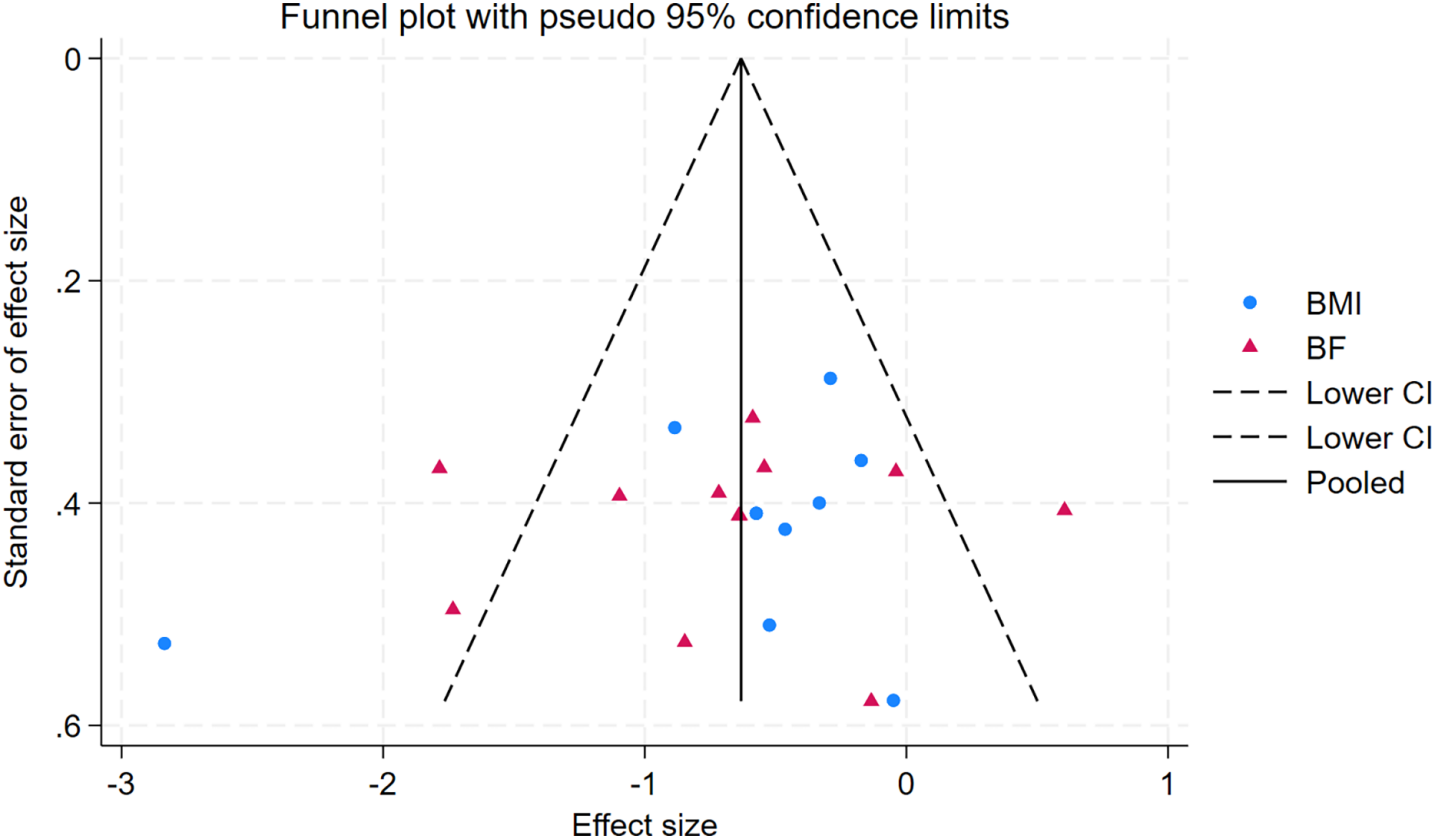
Funnel plot of body composition.

**Figure 14:**
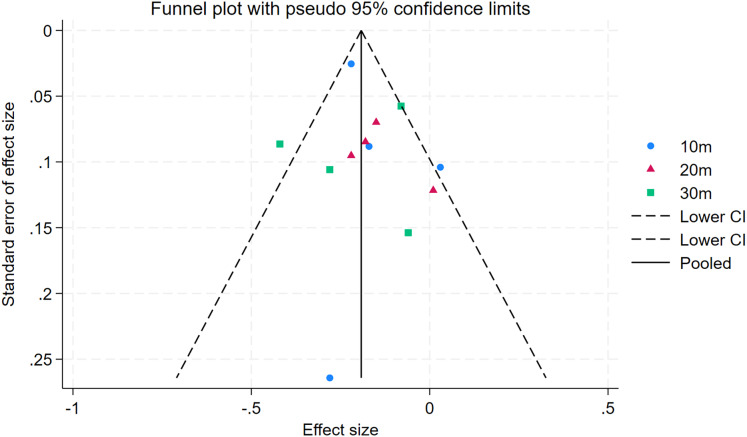
Funnel plot of sprint ability.

**Figure 15:**
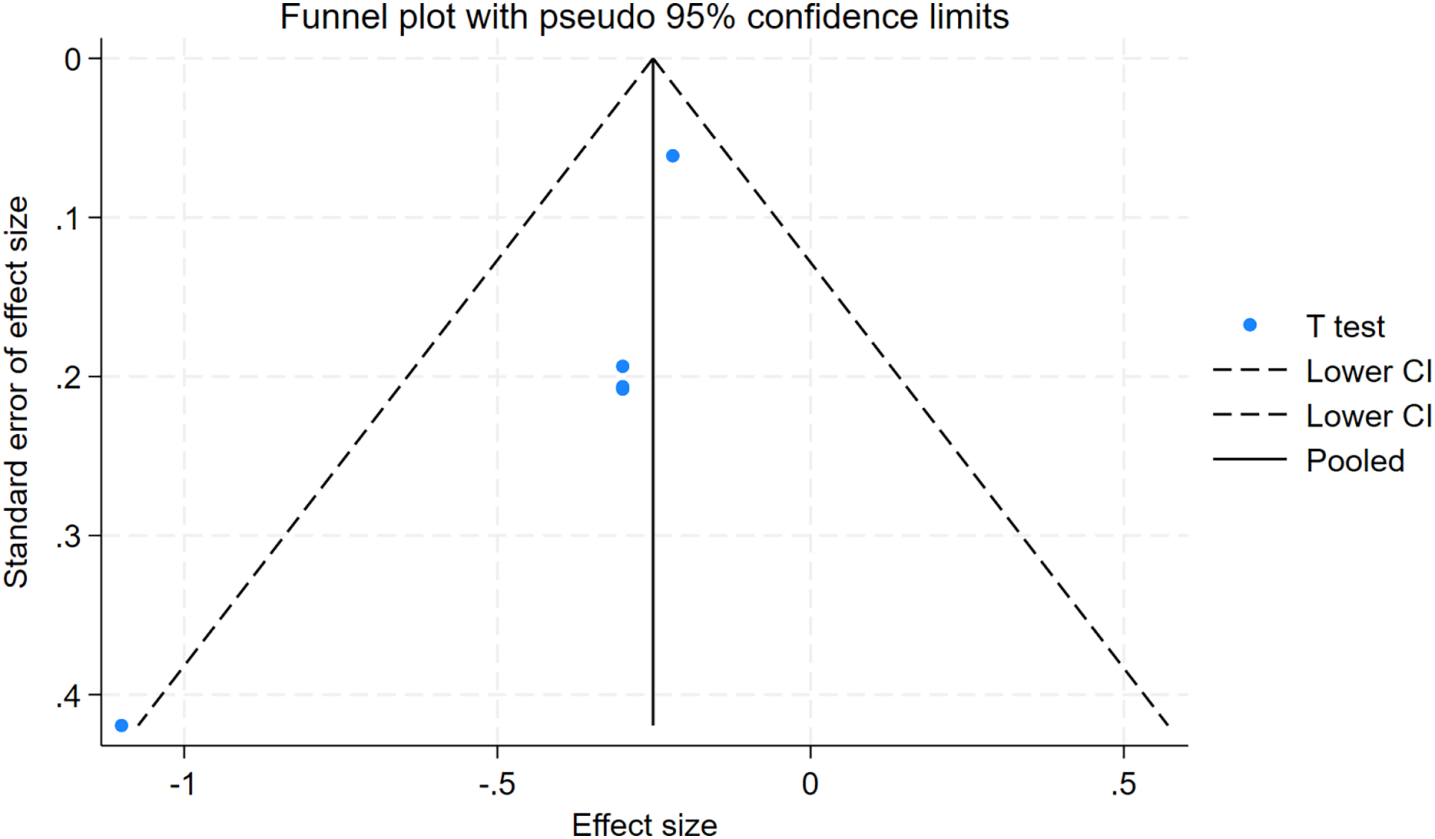
Funnel plot of change-of-direction ability.

**Figure 16:**
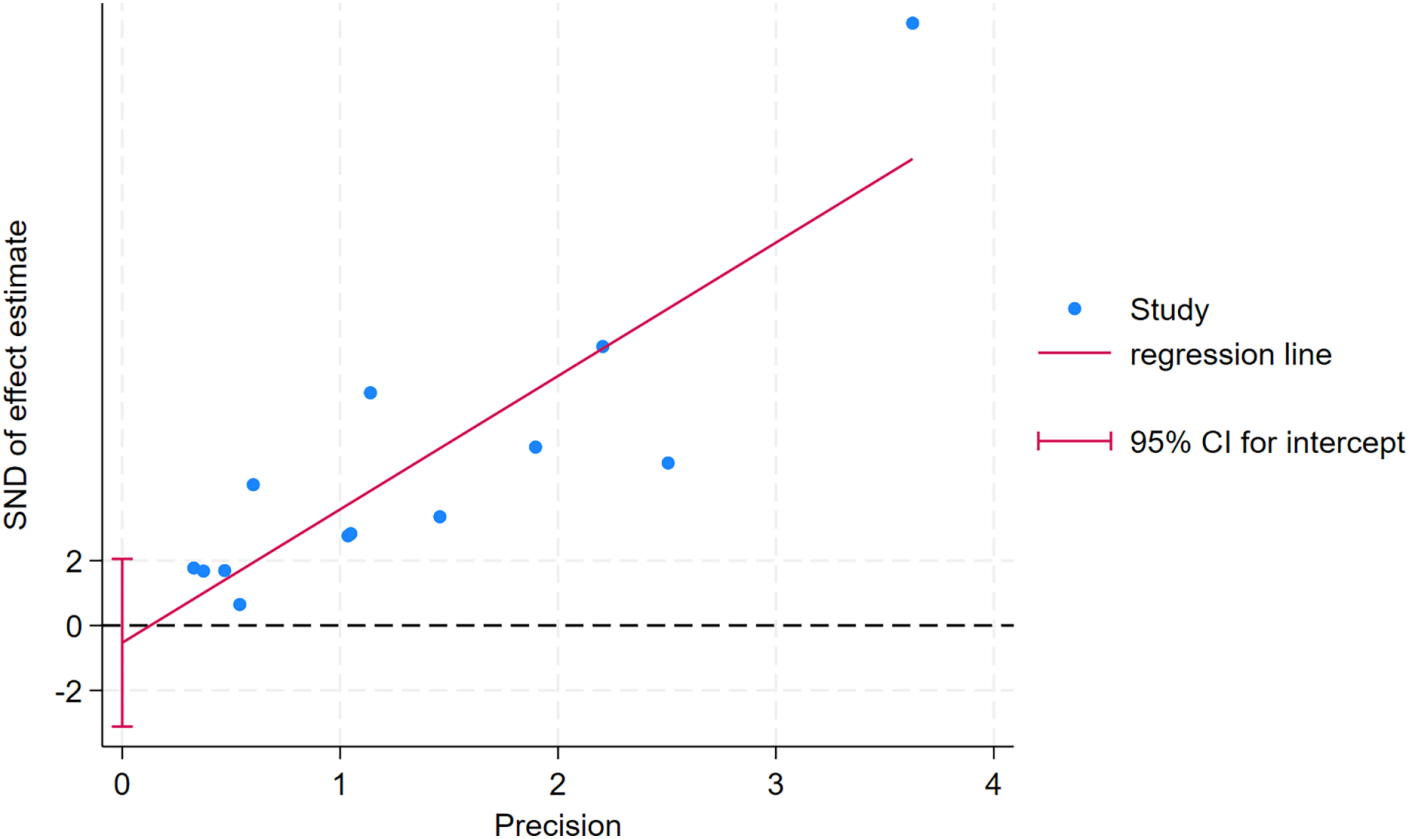
Egger’s test plots of aerobic capacity.

**Figure 17:**
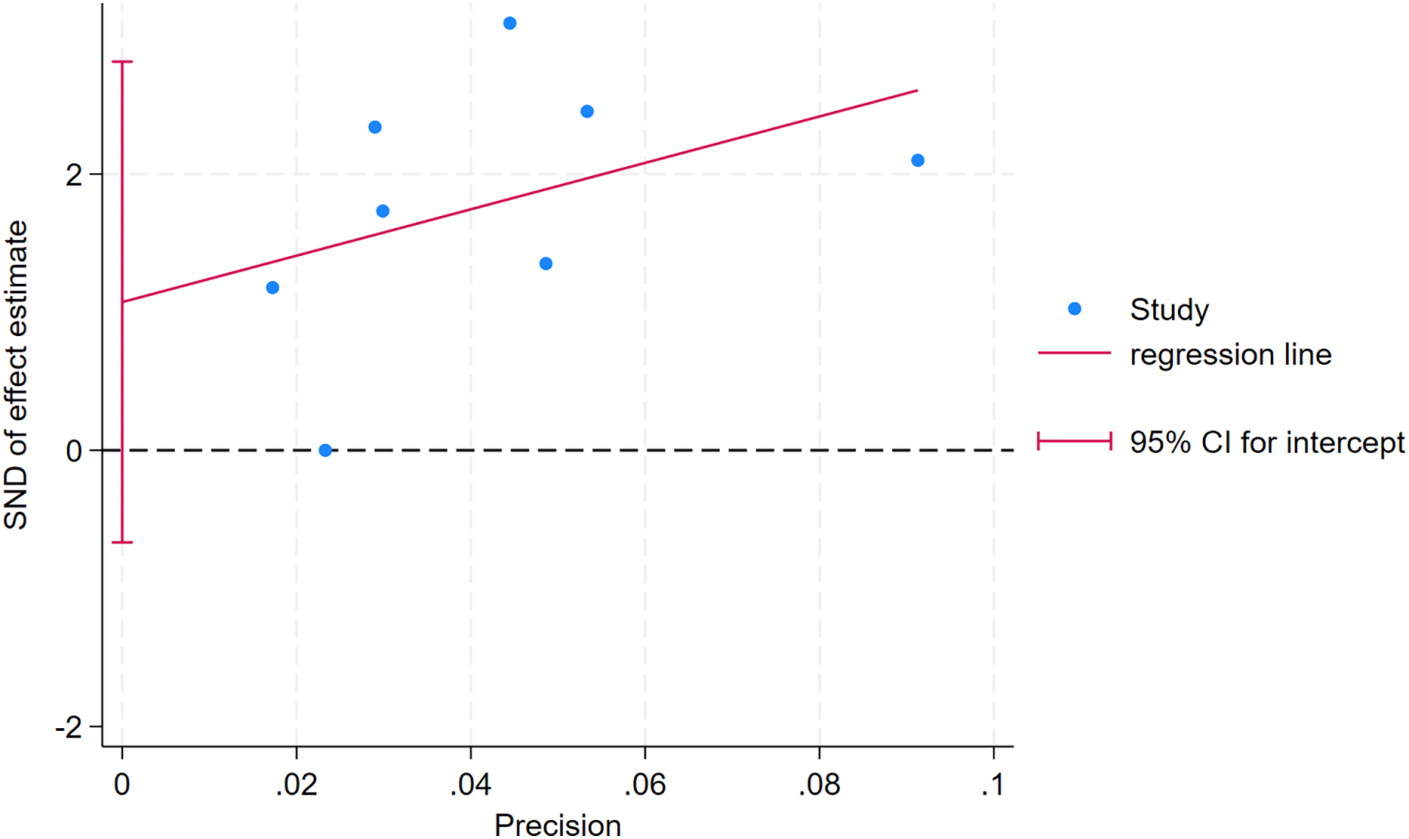
Egger’s test plots of anaerobic capacity.

**Figure 18:**
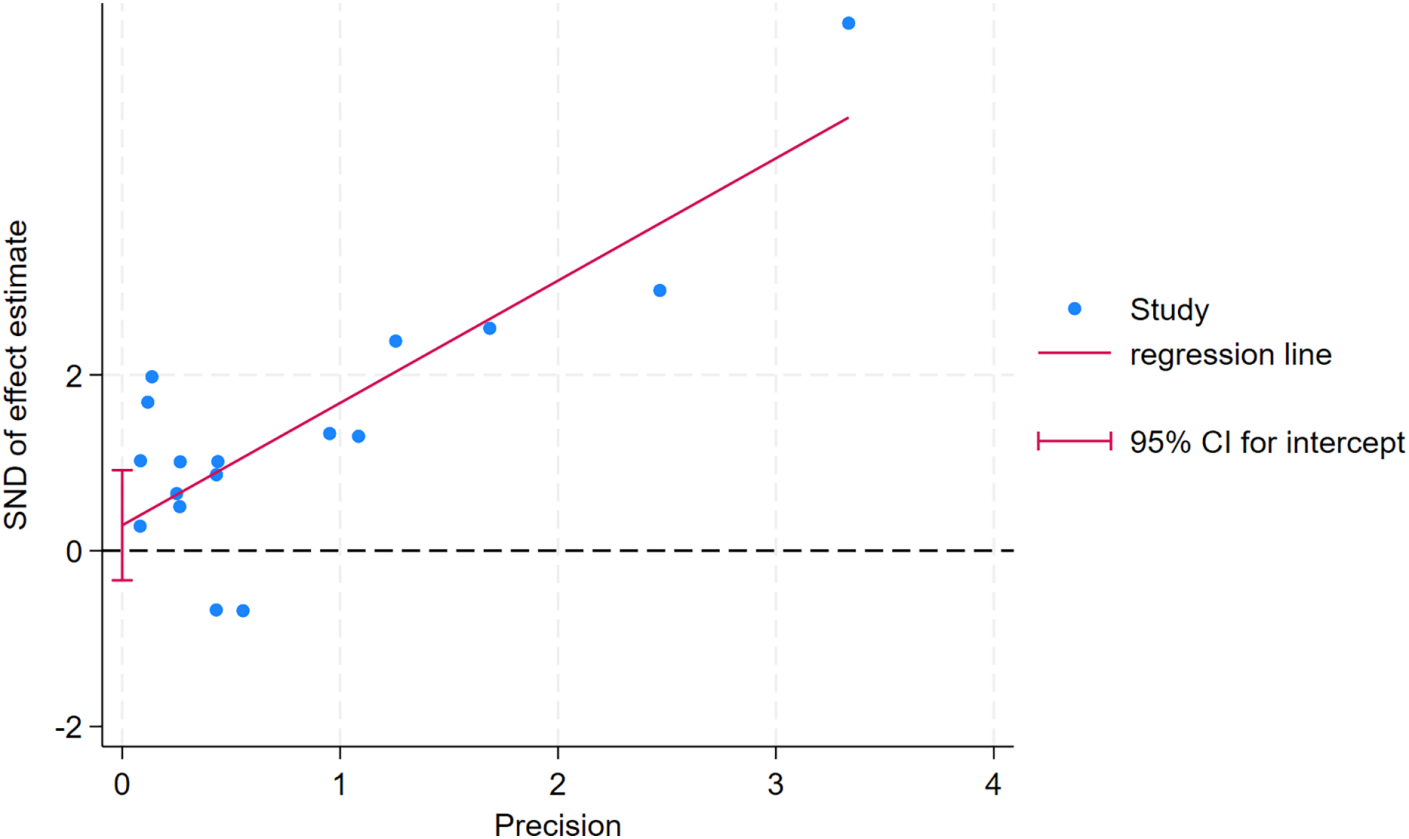
Egger’s test plots of jumping ability.

**Figure 19:**
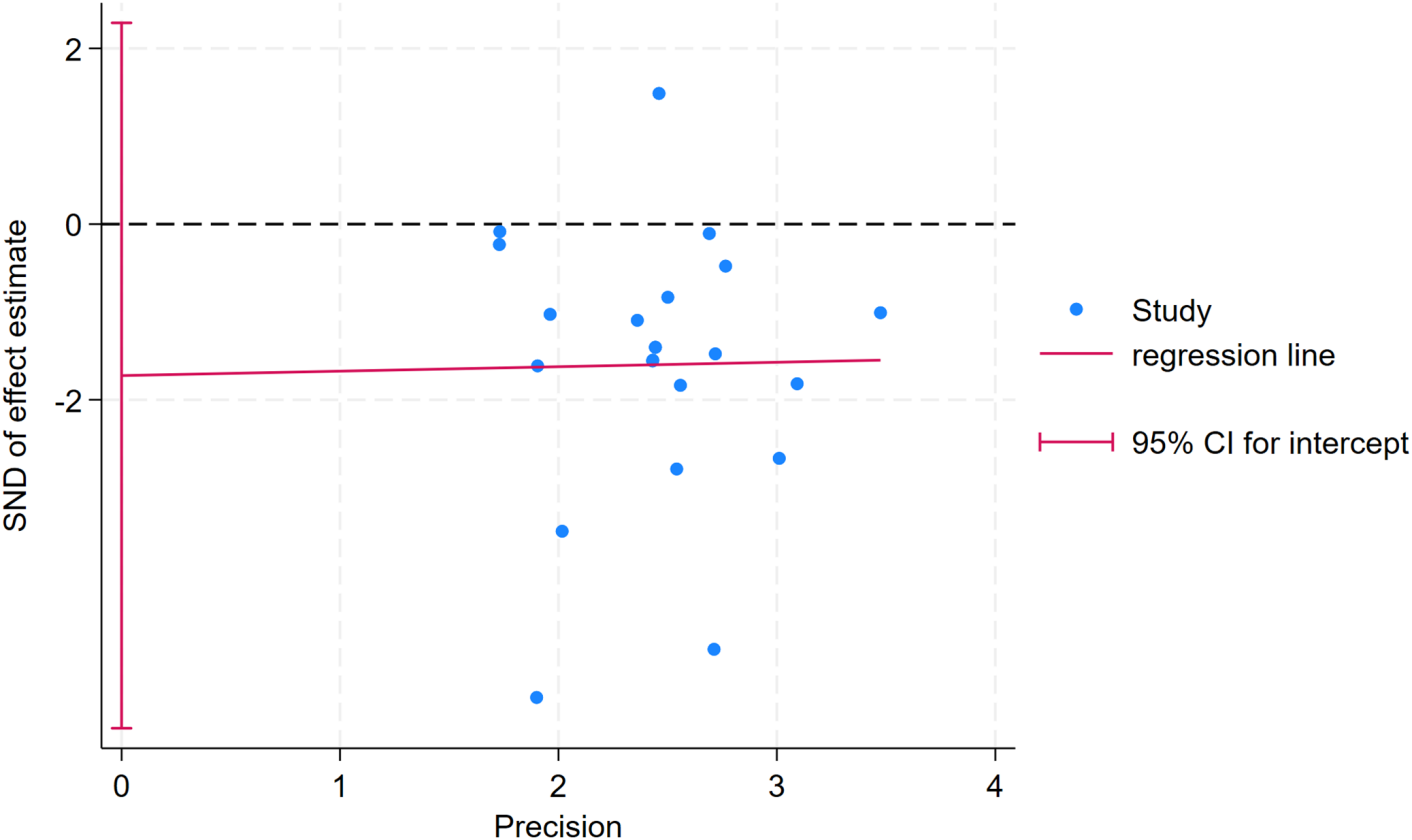
Egger’s test plots of body composition.

**Figure 20:**
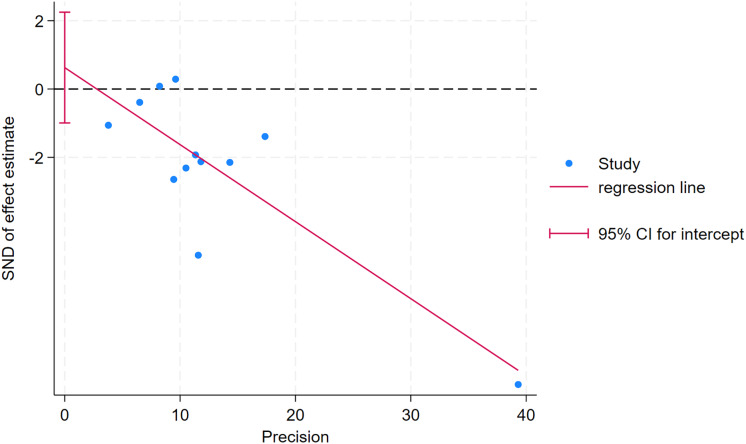
Egger’s test plots of sprint ability.

**Figure 21:**
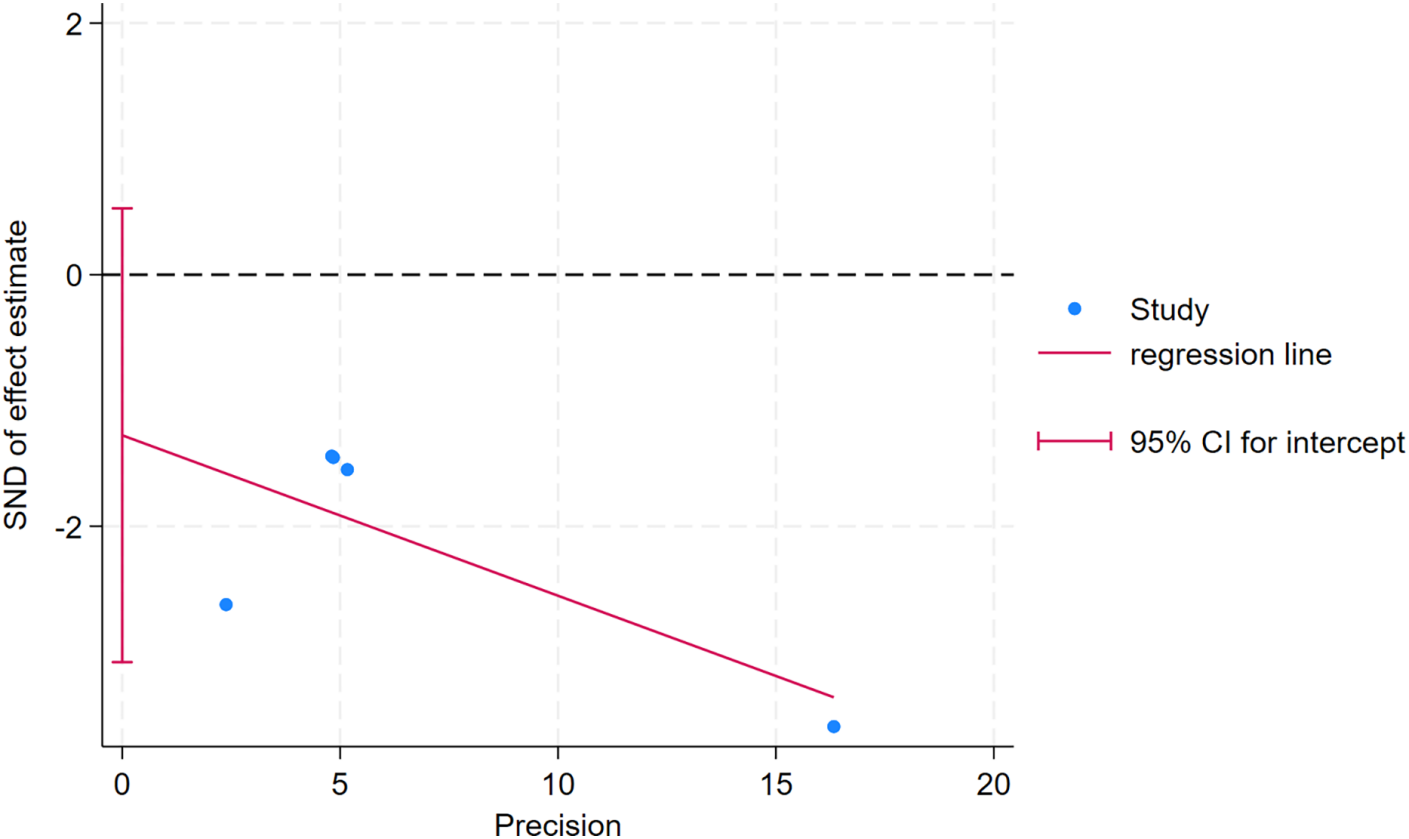
Egger’s test plots of change-of-direction ability.

As depicted in Figs. 10–15, visual inspection of funnel plots revealed slight asymmetry, suggesting a potential risk of publication bias. However, Egger’s regression test detected no evidence of significant publication bias for any outcome: aerobic capacity (t = −0.45, *p* = 0.660), anaerobic capacity (t = 1.51, *p* = 0.182), jumping ability (t = 0.99, *p* = 0.340), body composition (t = −0.90, *p* = 0.381), sprint ability (t = 0.86, *p* = 0.408), and change-of-direction ability (t = −2.25, *p* = 0.110) (Figs. 16–21). These results indicate that the pooled estimates are robust and unlikely to be materially distorted by publication bias.

### Sensitivity analysis

**Figure 22:**
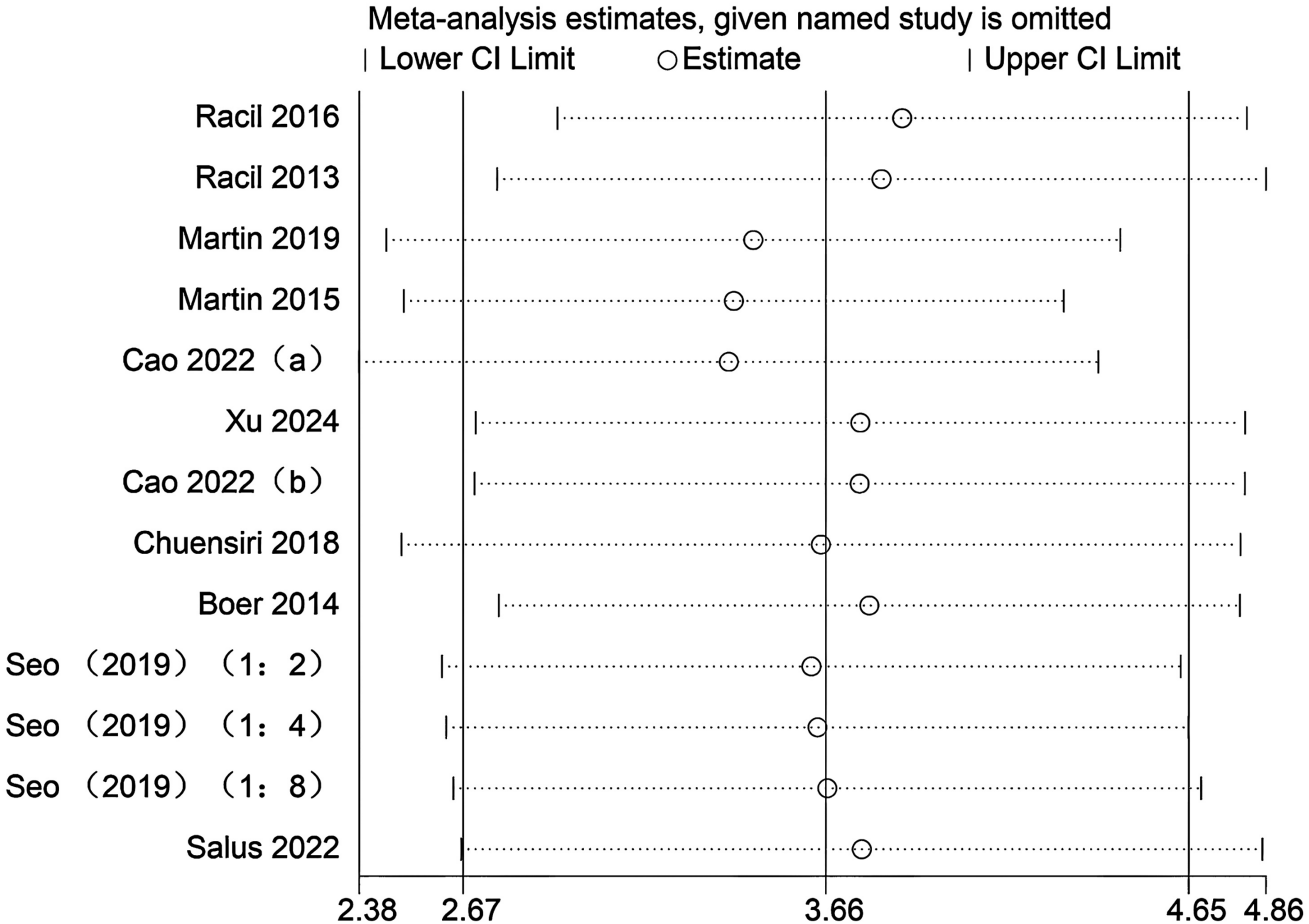
Sensitivity analysis of aerobic capacity (a/b, the same author published two articles in the same year; 1:2, SIT work-to-rest ratio is 1:2; 1:4, SIT work-to-rest ratio is 1:4; 1:8, SIT work-to-rest ratio is 1:8).

**Figure 23:**
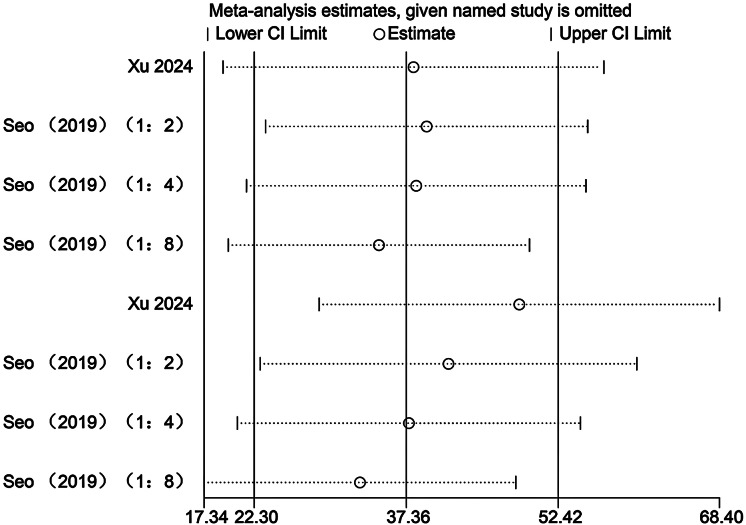
Sensitivity analysis of anaerobic capacity (1:2, SIT work-to-rest ratio is 1:2; 1:4, SIT work-to-rest ratio is 1:4; 1:8, SIT work-to-rest ratio is 1:8).

**Figure 24:**
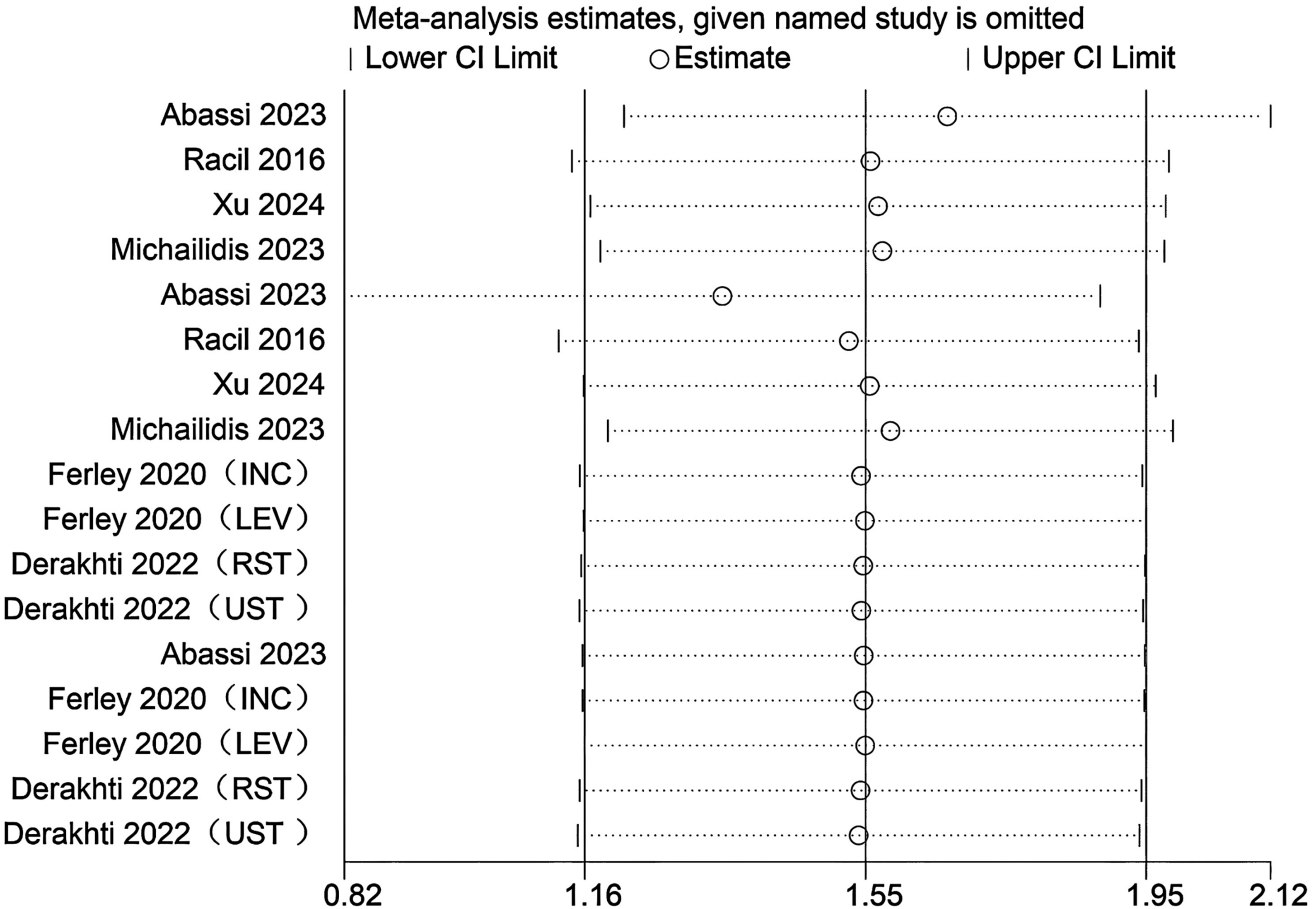
Sensitivity analysis of jumping ability (INC, SIT based on incline; LEV, SIT based on incline; RST, resisted sprint training; UST, unresisted sprint training).

**Figure 25:**
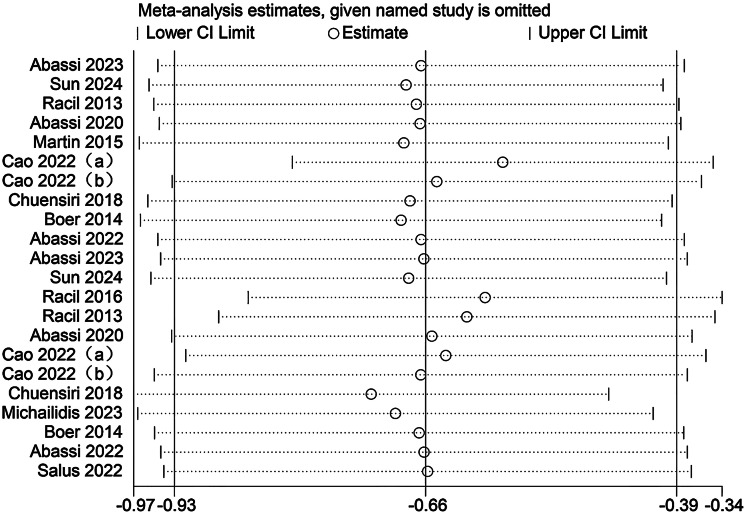
Sensitivity analysis of body composition (a/b, the same author published two articles in the same year).

**Figure 26:**
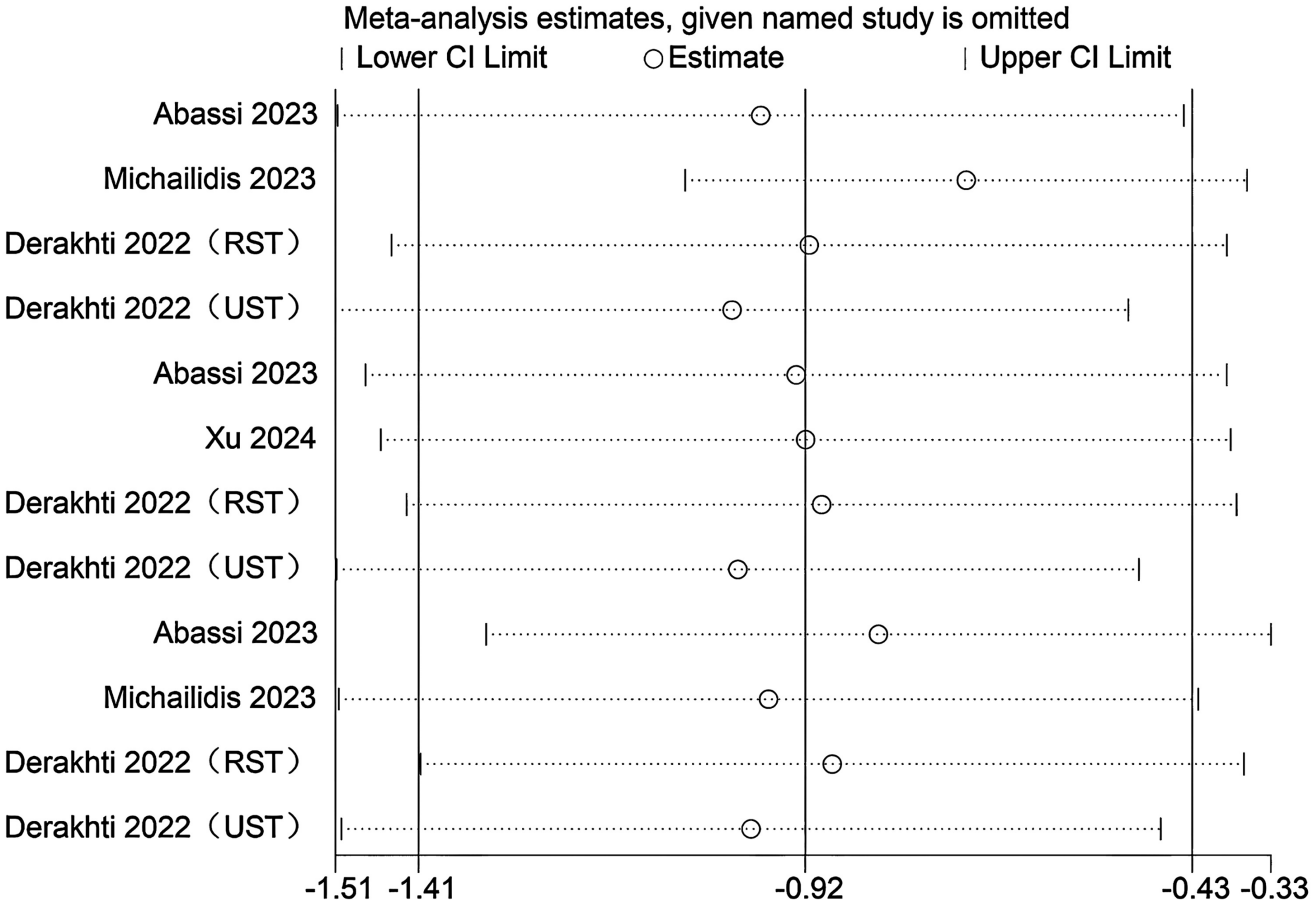
Sensitivity analysis of sprint ability (RST, resisted sprint training; UST, unresisted sprint training).

**Figure 27:**
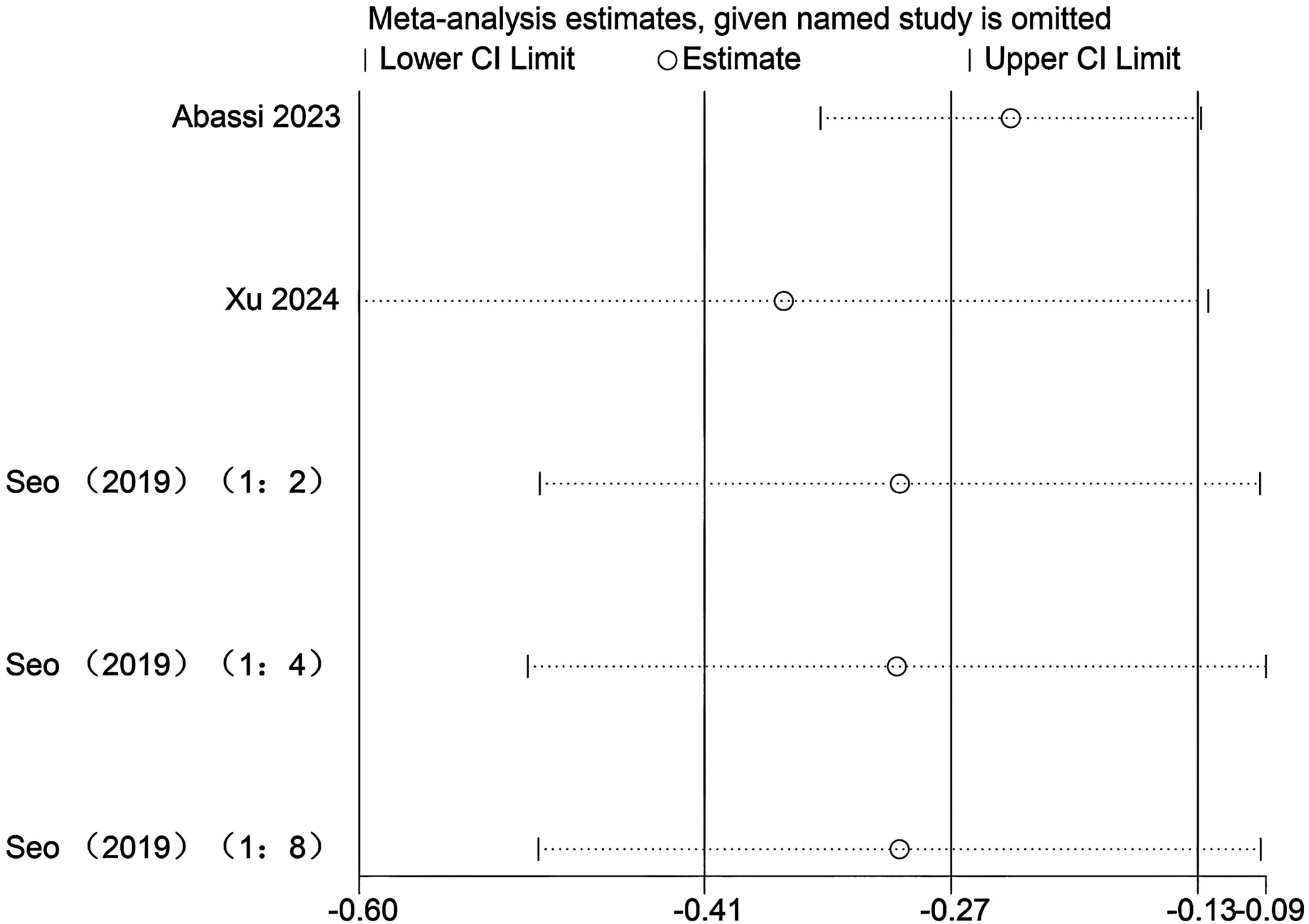
Sensitivity analysis of change-of-direction (1:2, SIT work-to-rest ratio is 1:2; 1:4, SIT work-to-rest ratio is 1:4; 1:8, SIT work-to-rest ratio is 1:8).

Sensitivity analyses were conducted to evaluate the potential influence of each individual study on the pooled meta-analytic results. As illustrated in Figs. 22–27, the pooled effect estimates remained stable: aerobic capacity fluctuated around 3.66, anaerobic capacity around 37.36, jumping ability around 1.55, body composition around −0.66, sprint ability around −0.92, and change-of-direction ability around −0.27. These observations indicated that the dataset of the present meta-analysis was robust.

## Discussion

### Aerobic capacity

The study results demonstrate that SIT is an effective strategy for enhancing the aerobic capacity of adolescents (VO₂ max, MD = 3.66). This finding is consistent with an extensive body of literature that has documented the ergogenic effects of SIT on cardiorespiratory fitness. In a meta-analysis, Gist et al. (2014) reported that SIT elicited a significant elevation in VO₂max in healthy adults, while requiring markedly lower exercise volumes than moderate-intensity continuous training (MICT) (ES = 0.32). Time constraints are consistently cited as the foremost barrier to regular exercise participation; integrating brief, high-intensity intervals within SIT therefore offers a time-efficient strategy to maximize physiological adaptations (Godin et al., 1994). Subsequent syntheses by Yang et al. (2021) (ES = 2.23) and Vasconcelos et al. (2020) (ES = 2.83) extended these observations to athletic cohorts, attributing the pronounced VO₂max gains to enhanced central circulatory function—namely, increased stroke volume and improved pulmonary gas-exchange efficiency. Adolescents, whose cardiopulmonary systems are still maturing, display baseline hemodynamic profiles that differ substantially from those of adults and trained athletes (Guohai, Liu & Xiaojian, 2016). By focusing exclusively on this age group, the current work provides targeted evidence that SIT is equally effective in augmenting VO₂max during adolescence.

Historically, the concept of “sensitive periods” in youth development has led to a de-emphasis on aerobic training during puberty, thereby limiting the scope of endurance-oriented interventions. Emerging evidence, however, indicates that adolescents retain a high degree of aerobic plasticity throughout pubertal progression. Mcmanus et al. (2005) observed significant VO₂max improvements in 10-year-old boys following 8 weeks of either interval or continuous training. Likewise, Yan, Kim & Choi (2022) documented substantial aerobic-capacity gains in 15-year-old athletes after a high-intensity interval training (HIIT) intervention. The trials included in the present review corroborate these observations, reporting VO₂max improvements across a broad spectrum of adolescent ages. The mechanistic basis of these improvements encompasses both central and peripheral adaptations. Peripheral adaptations include increased mitochondrial density and capillarization within skeletal muscle fibers, whereas central adaptations involve augmented stroke volume and cardiac output (Michailidis et al., 2023). Moreover, compared with continuous endurance exercise, SIT generates a transient intramuscular hypoxia that up-regulates PGC-1α mRNA expression, thereby enhancing oxidative metabolism and promoting mitochondrial biogenesis (Skovgaard, Almquist & Bangsbo, 2017).

Although the sensitivity analysis confirmed the robustness of the aerobic capacity results, substantial heterogeneity was observed in the overall effect, indicating that the findings should be interpreted with caution. The leave-one-out analysis revealed that the heterogeneity decreased to a moderate level after excluding the study by Martin et al. (2015). This may be attributed to the participant characteristics in that study. The participants were all healthy adolescents with normal body weight, a population with generally good physical function and metabolic levels, potentially allowing for more optimal adaptation to the high-intensity demands of SIT. In contrast, adolescents who are obese or overweight likely experience a greater physiological burden during SIT, and abnormalities in their metabolic and hormonal profiles might also influence the training response (Skovgaard, Almquist & Bangsbo, 2017). Conversely, adolescent athletes typically possess a superior baseline aerobic capacity and a strong adaptive response to training, which might explain the relatively smaller magnitude of improvement in aerobic capacity following SIT in this group. This inference is further supported by the results of the subgroup analysis. However, a notable observation is that for the other three outcomes subjected to subgroup analysis (jumping ability, body composition, and sprint ability), obese participants demonstrated more pronounced improvements compared to their non-obese counterparts. A potential explanation is that when performing the same high-intensity interval exercises, obese individuals must overcome a greater absolute load due to their higher body mass. This imposes a stronger stimulus on the neuromuscular system and results in higher energy expenditure, potentially leading to more significant training adaptations in terms of fat loss and enhanced force production (Browning & Kram, 2005).

### Anaerobic capacity

The present results revealed that SIT effectively enhanced adolescents’ anaerobic capacity (PP, MD = 48.39; AP, MD = 36.64). These findings substantially broaden the applicability of SIT beyond the adult and athletic populations that have dominated research to date. For example, Hall et al. (2023) meta-analytically confirmed a moderate effect of SIT on anaerobic capacity in healthy adults (ES = 0.61), whereas (Zhao et al., 2024) documented analogous benefits in competitive tennis players. The mechanistic rationale underlying these improvements centers on the “all-out” nature of SIT bouts, which acutely up-regulate creatine kinase and glycolytic enzyme activity, enhance intramuscular buffering capacity, and expand glycogen storage (Zhao et al., 2024). Collectively, these peripheral adaptations enable more rapid ATP resynthesis and more efficient energy utilization during subsequent anaerobic tasks, thereby elevating both PP and AP (Hall et al., 2023). Improvements in fatigue-related indices were further linked to the recovery-interval design of SIT; a recovery duration of ≥4 min is generally recommended to maximize glycolytic system contribution (Buchheit & Laursen, 2013).

By restricting inclusion to adolescent cohorts, the current synthesis addresses a critical gap in the literature. The observed low heterogeneity and high GRADE certainty underscore the robustness of these effects. Nevertheless, only two studies met the inclusion criteria, underscoring the paucity of data on SIT in adolescents despite compelling evidence in adults and athletes. As documented in the included trials, SIT aligns closely with adolescent physiological development, which is characterized by rapid somatic growth during puberty is accompanied by progressive increases in muscular strength, endurance, and explosive power (Seo et al., 2019; Sun et al., 2024). Short, high-intensity sprints provide a potent stimulus to the developing anaerobic energy system, while the challenging and varied nature of SIT sessions enhances motivation and engagement, further amplifying training adaptations in this age group (Seo et al., 2019; Sun et al., 2024).

### Jumping ability

The meta-analytic results showed that SIT had a significant positive effect on adolescents’ jumping ability (SJ, MD = 1.23; CMJ, MD = 1.74; SLJ, MD = 6.81). This outcome aligns with the majority of existing studies examining the relationship between SIT and jumping ability. For instance, Benítez-Flores et al. (2023) reported that SIT significantly improved SJ and CMJ scores in young women with obesity, and Lloria-Varella et al. (2023) observed a 5% increase in jump height after six SIT sessions in basketball players. The underlying mechanisms involve high-intensity sprint efforts that preferentially recruit fast-twitch fibers, enhance neuromuscular coordination, and increase the rate of force development, thereby augmenting lower-limb explosive power (Yang et al., 2024). In addition, SIT promotes muscle-fiber hypertrophy and neural adaptations that collectively improve jump performance (Estes et al., 2017). Conversely, some investigations have documented deteriorations in neuromuscular function and vagal reactivity after SIT, implying potential negative consequences for jump ability (Benítez-Flores et al., 2023). Among the trials included in the present review, Michailidis et al. (2023) observed reductions in both CMJ and SJ among adolescent soccer players after 4 weeks of SIT. This discrepancy may be attributed to the fact that jumping tasks primarily rely on the stretch shortening cycle (SSC), whereas SIT predominantly induces central and peripheral adaptations that may not directly translate into improved SSC performance (Buchheit & Laursen, 2013; Michailidis et al., 2023). Moreover, the 4-week duration employed by Michailidis et al. (2023) is shorter than that used in most other studies and may have been insufficient to elicit substantial neuromuscular adaptations, especially in adolescents whose responses are typically slower (Grădinaru & Oravițan, 2021).

To reconcile these disparate findings, the current meta-analysis quantitatively aggregated all available evidence and confirmed an overall beneficial effect of SIT on adolescent jumping ability. Several considerations may account for this positive outcome. First, duration response analyses indicate that interventions shorter than weeks yield only trivial gains, whereas programs exceeding 8 weeks produce marked enhancements (Schoenfeld et al., 2014). Extended SIT appears to facilitate greater motor-unit recruitment, refine intermuscular coordination, improve muscle synchrony, and diminish antagonist co activation adaptations that collectively maximize force output and explosive strength (Schoenfeld et al., 2014). Second, jumping tasks are predominantly fueled by the phosphagen and glycolytic energy systems; SIT has been shown to up-regulate both pathways, thereby enabling rapid force generation within the brief time frames characteristic of explosive movements (Okudaira et al., 2019). Since most participants in the studies included in this meta-analysis were overweight/obese adolescents, the observed improvements in jumping ability may be partly attributable to enhancements in anthropometric measures, such as weight reduction, resulting from the training intervention (da Silva et al., 2020; Racil et al., 2013). Furthermore, subgroup analysis results indicated that the training effect was superior in obese participants compared to non-obese participants, and greater improvements were observed in non-athlete populations than in athletes. These findings suggest a close relationship between baseline physical fitness and training adaptability: obese individuals may possess greater potential for improvement due to their initially lower fitness levels, while non-athlete populations might be more responsive to the intervention due to a lack of systematic training background (Delgado-Floody et al., 2020; Wetmore et al., 2020).

### Body composition

This study findings demonstrate that SIT significantly reduced both BMI and BFP in adolescents (BMI, SMD = −0.63; BFP, SMD = −0.68). A substantial literature already affirms that physical activity is a potent modulator of youth body composition, lowering BMI and BFP and conferring broad cardiometabolic benefits (Swift et al., 2014; Warburton & Bredin, 2017). Among available modalities, SIT—characterized by brief, supramaximal intermittent bouts—expends large amounts of energy in minimal time, acutely elevates fat oxidation, and chronically increases resting metabolic rate, thereby facilitating sustained reductions in adiposity and improvements in global health (Gillen et al., 2016). In a 12-week randomized trial, Hu et al. (2021) observed that SIT not only improved cardiorespiratory fitness and body composition in overweight young women but also enhanced exercise enjoyment and adherence. Subsequent work by Martin-Smith et al. (2019) and Salus et al. (2022) extended these findings to adolescents, demonstrating significant BMI and BFP reductions in both obese and healthy cohorts. Obesity is closely associated with multiple cardiometabolic diseases; overweight and obese adolescents are at greater risk of insulin resistance, type 2 diabetes, hypertension, and dyslipidemia (Kelishadi et al., 2015). Therefore, identifying effective exercise strategies to improve body composition and prevent chronic disease in youth is of critical importance.

By integrating multiple trials, the present meta-analysis corroborates the beneficial influence of SIT on adolescent body composition. These effects are shown to transcend simple elevations in energy expenditure and fat oxidation, implicating a multifactorial cascade that includes hormonal modulation and skeletal-muscle remodeling (Mølmen, Almquist & Skattebo, 2025; Song & Lan, 2024). Specifically, SIT acutely depletes muscle glycogen, which up-regulates insulin sensitivity and attenuates insulin resistance (Song & Lan, 2024). Concurrently, SIT augments skeletal-muscle oxidative capacity and mitochondrial density to a greater extent than either HIIT or MICT, thereby elevating resting metabolic rate and supporting long-term weight control (Mølmen, Almquist & Skattebo, 2025).

Nonetheless, conflicting findings have been reported. Chuensiri, Suksom & Tanaka (2018) reported no significant changes in BMI or BFP after SIT and hypothesized that compensatory increases in post-exercise appetite or energy intake neutralized the exercise-induced energy deficit. This possibility likely contributed to the considerable heterogeneity observed among the included body-composition studies. Furthermore, the regression analysis identified the type of SIT as a source of heterogeneity. This may be attributed to differences in the muscle groups recruited, energy metabolic pathways involved, and mechanical loading characteristics across different exercise modalities, potentially leading to inconsistent effects on body composition (Poon et al., 2024). These findings are corroborated by further subgroup analyses. Another meta-analysis has indicated that high-intensity training did not significantly alter BMI or BFP in combat sports athletes, a result potentially linked to the relatively short intervention duration (Vasconcelos et al., 2020). Concurrently, while high-intensity training is sufficient to modify BMI and BFP in overweight and obese individuals, well-trained adolescent athletes typically already exhibit low baseline levels of these metrics. Consequently, SIT interventions may produce minimal further discernible changes in these variables (Batacan et al., 2017; Franchini, Brito & Artioli, 2012; Stoggl & Sperlich, 2014). Nevertheless, even after accounting for these factors in the regression analysis, a residual heterogeneity of 50% persisted. This suggests that the observed heterogeneity may also stem from other confounding factors not captured in our model. Therefore, final conclusions regarding the effect of SIT on body composition in adolescents must be drawn with considerable caution.

### Sprint ability

Meta-analytic results indicated that SIT significantly improved adolescents’ sprint ability (10 m, MD = −0.16; 20 m, MD = −0.15; 30 m, MD = −0.22). These findings are consistent with most previous studies on the effect of SIT on sprint speed. For example, Litleskare et al. (2020) observed pronounced reductions in 60-m sprint times in healthy adults after SIT, attributing the gains to marked elevations in peak power output. Similarly, Pérez-Ifrán et al. (2024) reported significant improvements in 5 and 10 m sprint performance after only five SIT sessions. These benefits were mediated by increased muscle buffering capacity, up-regulated anaerobic enzyme activity, and diminished metabolic and ionic perturbations during high-intensity efforts (Pérez-Ifrán et al., 2024). In addition, peripheral skeletal-muscle adaptations elicited by SIT may represent another underlying mechanism for the observed speed gains. This meta-analysis, synthesizing previous research, confirms that SIT significantly enhances sprint speed in adolescents, consistent with most included studies. The mechanisms underpinning SIT-induced improvements in jumping and anaerobic performance likely also apply to sprint speed. Specifically, SIT preferentially recruits type II fast-twitch fibers and increases their firing frequency, enhancing both morphological and neural adaptations of the lower-limb musculature, thereby augmenting lower-limb force production and, ultimately, sprint speed (Abassi et al., 2023). Moreover, the high degree of task specificity between training content and sprint action contributes to performance gains; repeated high-intensity sprint bouts enable greater neuromuscular adaptation to the movement pattern, resulting in superior sprint times (Racil et al., 2013). Improvements in sprint velocity may also arise from enhanced lower-limb intermuscular coordination and increased step frequency (Buchheit et al., 2010).

Nevertheless, some studies have reported contrasting findings. Buchheit et al. (2010) observed no significant improvements in acceleration or repeated-sprint ability following a 4-week SIT intervention in highly trained, in-season handball athletes. The absence of measurable gains likely reflects a ceiling effect: athletes already exposed to intensive sport-specific training possess elevated baseline neuromuscular and metabolic capacities, rendering the additional SIT stimulus insufficient to elicit further adaptation (Buchheit et al., 2010). Additionally, the athletes’ high baseline training status may have blunted the training response, preventing the SIT protocol from imposing sufficient novel load on the neuromuscular system to enhance maximal running speed (Ross, Leveritt & Riek, 2001). Although sensitivity analyses confirmed the overall robustness of the pooled estimates, moderate heterogeneity persisted. Stepwise exclusion identified two primary sources of variability: (1) Abassi et al. (2023) recruited obese adolescents, whereas the remaining studies enrolled athletic youth; and (2) Derakhti et al. (2022) employed a 4-week intervention, whereas all other protocols lasted at least 6 weeks. These methodological and sample-related differences are likely contributors to the observed heterogeneity. Furthermore, the examined covariates could not fully account for the heterogeneity, suggesting potential influences from unmeasured confounding factors, statistical power limitations, and measurement discrepancies. Consequently, these findings should be interpreted with caution.

### Change-of-direction ability

This study findings demonstrated that SIT significantly improved adolescents’ change-of-direction ability (T test, MD = −0.27). To date, only a handful of studies have examined the impact of SIT on change-of-direction. The present meta-analysis is the first to focus specifically on adolescents, thereby extending the evidence base for this population. The findings align with those of Song & Jilikeha (2023), who reported that SIT—by augmenting lower-limb explosive power and neuromuscular efficiency—optimizes the braking–reacceleration sequence required during rapid directional changes, thereby enhancing basketball-specific change-of-direction. Yan & Li (2025) further demonstrated that SIT promotes preferential recruitment of type II fibers and facilitates rapid eccentric–concentric transitions within minimal ground-contact times, translating to improved change-of-direction performance in adolescent soccer players.

Although the present meta-analysis revealed low heterogeneity among the included change-of-direction ability studies, only five trials were available, and all utilized the T test as the sole outcome measure. This limited scope suggests that, while SIT appears beneficial, the observed effect size could be overestimated due to small sample size or narrow measurement dimensions. Notably, a recent study of adolescent soccer players found that 8 weeks of SIT improved both illinois and T test scores. However, compared with a speed agility quickness (SAQ) program, SIT conferred advantages primarily in neural conduction velocity and sprint speed, whereas gains in change-of-direction requiring complex movement patterns were more modest (Duan, Kim & Park, 2024). Similarly, Xu et al. (2024) reported smaller T test improvements in basketball players following SIT than after small-sided games (SSG), attributing the discrepancy to the richer repertoire of sport-specific change-of-direction patterns and more ecologically valid stimuli provided by SSG. Collectively, these data indicate that SIT is an effective stimulus for enhancing adolescent change-of-direction ability, yet its benefits are maximized when integrated with sport-specific change-of-direction drills and neuromuscular-control exercises to ensure comprehensive development of multidirectional movement qualities in youth athletes.

### Limitations and prospects

This study systematically evaluated the effects of SIT on adolescents’ physical fitness, yet several limitations warrant consideration. Firstly, to ensure retrieval and data extraction accuracy, this review exclusively included English-language publications. Although English serves as the primary medium for scientific communication and most research in this field is published in English, this approach may have excluded potentially relevant studies in other languages, thereby introducing potential language bias. Secondly, substantial heterogeneity was observed for certain outcome measures. Although meta-regression analyses were conducted to explore specific covariates, they failed to fully explain the heterogeneity sources, suggesting that other unknown or unmeasured confounding factors might have influenced effect sizes. Consequently, these findings require cautious interpretation. Furthermore, the limited number of included studies, particularly regarding anaerobic capacity and change-of-direction outcomes, increases the potential risk of false-positive results in the meta-analysis. Finally, considering adolescents’ significant variations in physical development, body composition, and motor capabilities across different pubertal stages, along with corresponding differences in SIT adaptability, while most participants in included studies were late-pubertal adolescents, this restricted deeper investigation into age-specific SIT training effects through subgroup analyses.

Based on these limitations, future research should develop along these directions: First, when resources permit, systematically search Chinese and Western academic databases and collaborate with multilingual teams to establish a more comprehensive global evidence base and minimize language bias. Second, expand sample sizes to systematically examine SIT’s effects on various physical performance indicators in adolescents, thereby obtaining more comprehensive evidence. Third, regarding heterogeneity sources, future primary studies should standardize reporting of key covariates like participant baseline characteristics and training adherence, providing essential data foundations for subsequent comprehensive analyses. Fourth, future primary research should closely integrate adolescent physical development models, explicitly distinguishing early-, mid-, and late-pubertal participants during experimental design phases. This approach will generate necessary data for exploring developmental stage-specific SIT effects and facilitate formulated precision exercise prescriptions.

## Conclusions

SIT elicits significant improvements in adolescents’ aerobic capacity, anaerobic power, jumping ability, body composition, sprint ability, and change-of-direction ability. Collectively, these findings establish SIT as a potent, time-efficient intervention for comprehensively enhancing youth physical fitness.

### Practical applications

SIT has demonstrated significant potential in enhancing physical fitness among adolescents and can be applied across various domains of youth sports. In school physical education practice, its time-efficient nature makes it particularly suitable for addressing the current challenges of heavy academic workloads and prolonged sedentary behavior among adolescents. Physical education teachers can incorporate SIT models based on “all-out sprints followed by active recovery” into regular curricula. This approach is not only easy to organize and implement but also contribute to rapid improvements in body composition and cardiorespiratory health. For adolescent athletes, SIT serves as an effective method for developing physical conditioning. Coaches may schedule 1–2 SIT sessions per week during preparatory or pre-competition intensification phases. However, it should be noted that our findings suggest SIT may have limited effects on improving agility involving complex decision-making and multi-directional movements. Therefore, to maximize athletic performance gains, it is recommended to combine SIT with agility training that incorporates sport-specific movement patterns, thereby more comprehensively addressing the demands of specific sports.

Furthermore, safety and individualization should be prioritized during implementation. Given that adolescents’ musculoskeletal systems are not fully mature, adequate warm-up must be conducted before training, with emphasis placed on correct movement techniques and landing mechanics to prevent sports injuries. Training load should follow the principle of progressive overload: intensity may be appropriately reduced or sprint duration shortened in the initial phase, and gradually increased as adaptation occurs. Additionally, individual differences must be taken into account to ensure the safety and effectiveness of the training program.

## Supplemental Information

10.7717/peerj.21252/supp-1 Supplemental Information 1 Inclusion and Exclusion of the Literature.

10.7717/peerj.21252/supp-2 Supplemental Information 2 Search strategy.

10.7717/peerj.21252/supp-3 Supplemental Information 3 PRISMA checklist.

10.7717/peerj.21252/supp-4 Supplemental Information 4 Raw data.

## Additional Information and Declarations

### Data Availability

The following information was supplied regarding data availability:

This is a systematic review/meta-analysis.
