# Supplementary material for: Effectiveness of sprint interval training in enhancing adolescent physical fitness: a systematic review and meta-analysis
Source: PeerJ. 2026 Jun 2;14:e21252. doi: 10.7717/peerj.21252 (PMC13239486; doi:10.7717/peerj.21252)
Supplement: Supplemental Information 2 [file peerj-14-21252-s002.docx]

| Database | Web of Science | |
| --- | --- | --- |
| Search Date | June 2, 2025 | |
| Search Period | From the inception of database to June 2, 2025 | |
| No. | Search strategy | Literatures retrieved |
| #1 | AB=("sprinting training" OR "sprint-interval" OR "sprint exercise" OR "sprint interval training" OR "SIT" OR "high intensity interval training" OR "HIIT") | 29,090 |
| #2 | AB=("physical performance" OR "athletic performance" OR "power" OR "strength" OR "force" OR "speed" OR "jump" OR "velocity" OR "output" OR "explosive strength" OR "explosive force" OR "explosive effort" OR "explosive power" OR "bursting force" OR "explosive strength" OR "sprint" OR "explosiveness" OR "agility" OR "Change of direction" OR "aerobic capacity" OR "aerobic ability" OR "aerobic performance" OR "maximal oxygen uptake" OR "aerobic endurance" OR "anaerobic capacity" OR "anaerobic ability" OR "anaerobic capability" OR "anaerobic power" OR "anaerobic endurance" OR "endurance") | 6,868,108 |
| #3 | AB=("adolescent" OR "teenager" OR "youth" OR "youngsters" OR "adolescent students" OR "adolescents" OR "child" OR "children" OR "student" OR "students" OR "female adolescent" OR "male adolescent" OR "young athletes" OR "adolescent athletes") | 2,463,862 |
| #4 | #3 AND #2 AND #1 | 1349 |

| Database | PubMed | |
| --- | --- | --- |
| Search Date | June 2, 2025 | |
| Search Period | From the inception of database to June 2, 2025 | |
| No. | Search strategy | Literatures retrieved |
| #1 | ("sprinting training"[Title/Abstract]) OR ("sprint-interval"[Title/Abstract]) OR ("sprint exercise"[Title/Abstract]) OR ("sprint interval training"[Title/Abstract]) OR ("SIT"[Title/Abstract]) OR ("high intensity interval training"[Title/Abstract]) OR ("HIIT"[Title/Abstract])) | 21,839 |
| #2 | ("physical performance"[Title/Abstract]) OR ("athletic performance"[Title/Abstract]) OR ("power"[Title/Abstract]) OR ("strength"[Title/Abstract]) OR ("force"[Title/Abstract]) OR ("speed"[Title/Abstract]) OR ("jump"[Title/Abstract]) OR ("velocity"[Title/Abstract]) OR ("output"[Title/Abstract]) OR ("explosive strength"[Title/Abstract]) OR ("explosive force"[Title/Abstract]) OR ("explosive effort"[Title/Abstract]) OR ("explosive power"[Title/Abstract]) OR ("bursting force"[Title/Abstract]) OR ("explosive strength"[Title/Abstract]) OR ("sprint"[Title/Abstract]) OR ("explosiveness"[Title/Abstract]) OR ("agility"[Title/Abstract]) OR ("Change of direction"[Title/Abstract]) OR ("aerobic capacity"[Title/Abstract]) OR ("aerobic ability"[Title/Abstract]) OR ("aerobic performance"[Title/Abstract]) OR ("maximal oxygen uptake"[Title/Abstract]) OR ("aerobic endurance"[Title/Abstract]) OR ("anaerobic capacity"[Title/Abstract]) OR ("anaerobic ability"[Title/Abstract]) OR ("anaerobic capability"[Title/Abstract]) OR ("anaerobic power"[Title/Abstract]) OR ("anaerobic endurance"[Title/Abstract]) OR ("endurance"[Title/Abstract]) | 1,806,084 |
| #3 | ("adolescent"[Title/Abstract]) OR ("teenager"[Title/Abstract]) OR ("youth"[Title/Abstract]) OR ("youngsters"[Title/Abstract]) OR ("adolescent students"[Title/Abstract]) OR ("adolescents"[Title/Abstract]) OR ("child"[Title/Abstract]) OR ("children"[Title/Abstract]) OR ("student"[Title/Abstract]) OR ("students"[Title/Abstract]) OR ("female adolescent"[Title/Abstract]) OR ("male adolescent"[Title/Abstract]) OR ("young athletes"[Title/Abstract]) OR ("adolescent athletes"[Title/Abstract]) | 2,251,976 |
| #4 | #3 AND #2 AND #1 | 1149 |

| Database | MEDLINE | |
| --- | --- | --- |
| Search Date | June 2, 2025 | |
| Search Period | From the inception of database to June 2, 2025 | |
| No. | Search strategy | Literatures retrieved |
| #1 | AB("sprinting training" OR "sprint-interval" OR "sprint exercise" OR "sprint interval training" OR "SIT" OR "high intensity interval training" OR "HIIT") | 20,413 |
| #2 | AB("physical performance" OR "athletic performance" OR "power" OR "strength" OR "force" OR "speed" OR "jump" OR "velocity" OR "output" OR "explosive strength" OR "explosive force" OR "explosive effort" OR "explosive power" OR "bursting force" OR "explosive strength" OR "sprint" OR "explosiveness" OR "agility" OR "Change of direction" OR "aerobic capacity" OR "aerobic ability" OR "aerobic performance" OR "maximal oxygen uptake" OR "aerobic endurance" OR "anaerobic capacity" OR "anaerobic ability" OR "anaerobic capability" OR "anaerobic capacity" OR "anaerobic power" OR "anaerobic endurance" OR "endurance") | 1,709,923 |
| #3 | AB("adolescent" OR "teenager" OR "youth" OR "youngsters" OR "adolescent students" OR "adolescents" OR "child" OR "children" OR "student" OR "students" OR "female adolescent" OR "male adolescent" OR "young athletes" OR "adolescent athletes") | 1,769,234 |
| #4 | #3 AND #2 AND #1 | 1054 |

| Database | Cochrane Library | |
| --- | --- | --- |
| Search Date | June 2, 2025 | |
| Search Period | From the inception of database to June 2, 2025 | |
| No. | Search strategy | Literatures retrieved |
| #1 | Abstract("sprinting training" OR "sprint-interval" OR "sprint exercise" OR "sprint interval training" OR "SIT" OR "high intensity interval training" OR "HIIT") | 21,008 |
| #2 | Abstract("physical performance" OR "athletic performance" OR "power" OR "strength" OR "force" OR "speed" OR "jump" OR "velocity" OR "output" OR "explosive strength" OR "explosive force" OR "explosive effort" OR "explosive power" OR "bursting force" OR "explosive strength" OR "sprint" OR "explosiveness" OR "agility" OR "Change of direction" OR "aerobic capacity" OR "aerobic ability" OR "aerobic performance" OR "maximal oxygen uptake" OR "aerobic endurance" OR "anaerobic capacity" OR "anaerobic ability" OR "anaerobic capability" OR "anaerobic power" OR "anaerobic endurance" OR "endurance") | 194,842 |
| #3 | Abstract("adolescent" OR "teenager" OR "youth" OR "youngsters" OR "adolescent students" OR "adolescents" OR "child" OR "children" OR "student" OR "students" OR "female adolescent" OR "male adolescent" OR "young athletes" OR "adolescent athletes") | 196,734 |
| #4 | #3 AND #2 AND #1 | 1019 |
